# Supplementary material for: A haplotype-based evolutionary history of barley domestication
Source: Nature. 2025 Sep 24;647(8090):680–8. doi: 10.1038/s41586-025-09533-7 (PMC12629985; doi:10.1038/s41586-025-09533-7)
Supplement: Supplementary file 1 — Supplementary Figures 1–30 [file 41586_2025_9533_MOESM1_ESM.pdf]

nature portfolio

https://doi.org/10.1038/s41586-025-09533-7

Supplementary information

A haplotype-based evolutionary history of barley domestication

In the format provided by the authors and unedited

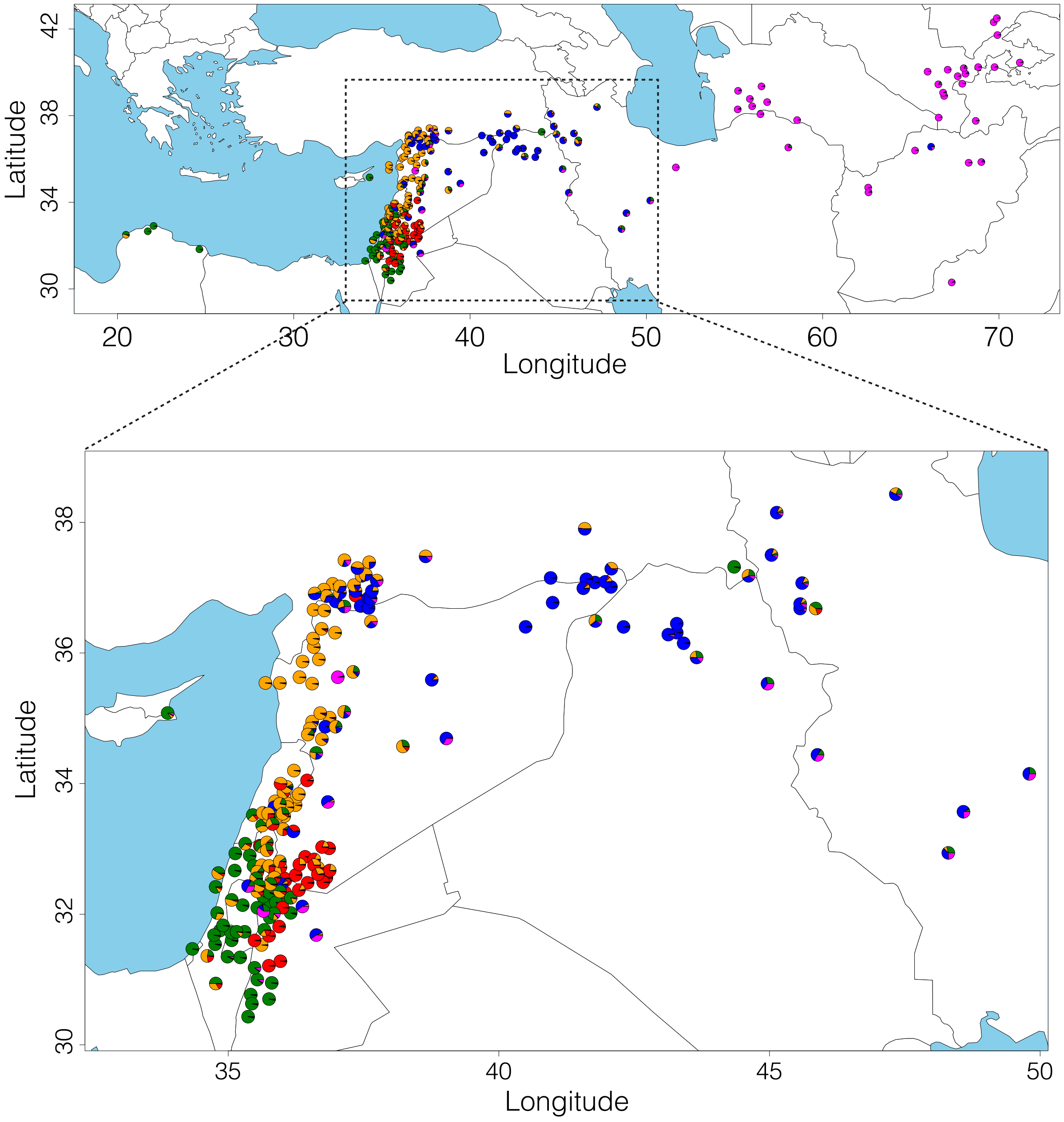

Supplementary Figure 1: ancestry composition of wild barley populations based on Admixture analysis with *K* = 5. Each pie chart represents the ancestral proportions of a single sample, plotted at its corresponding geographic location using latitude and longitude coordinates. Only samples with geographic information (n = 237) are shown on the map. Geographic outlines were obtained from the R package 'maps' (https://CRAN.R-project.org/package=maps), which uses public-domain base map data.

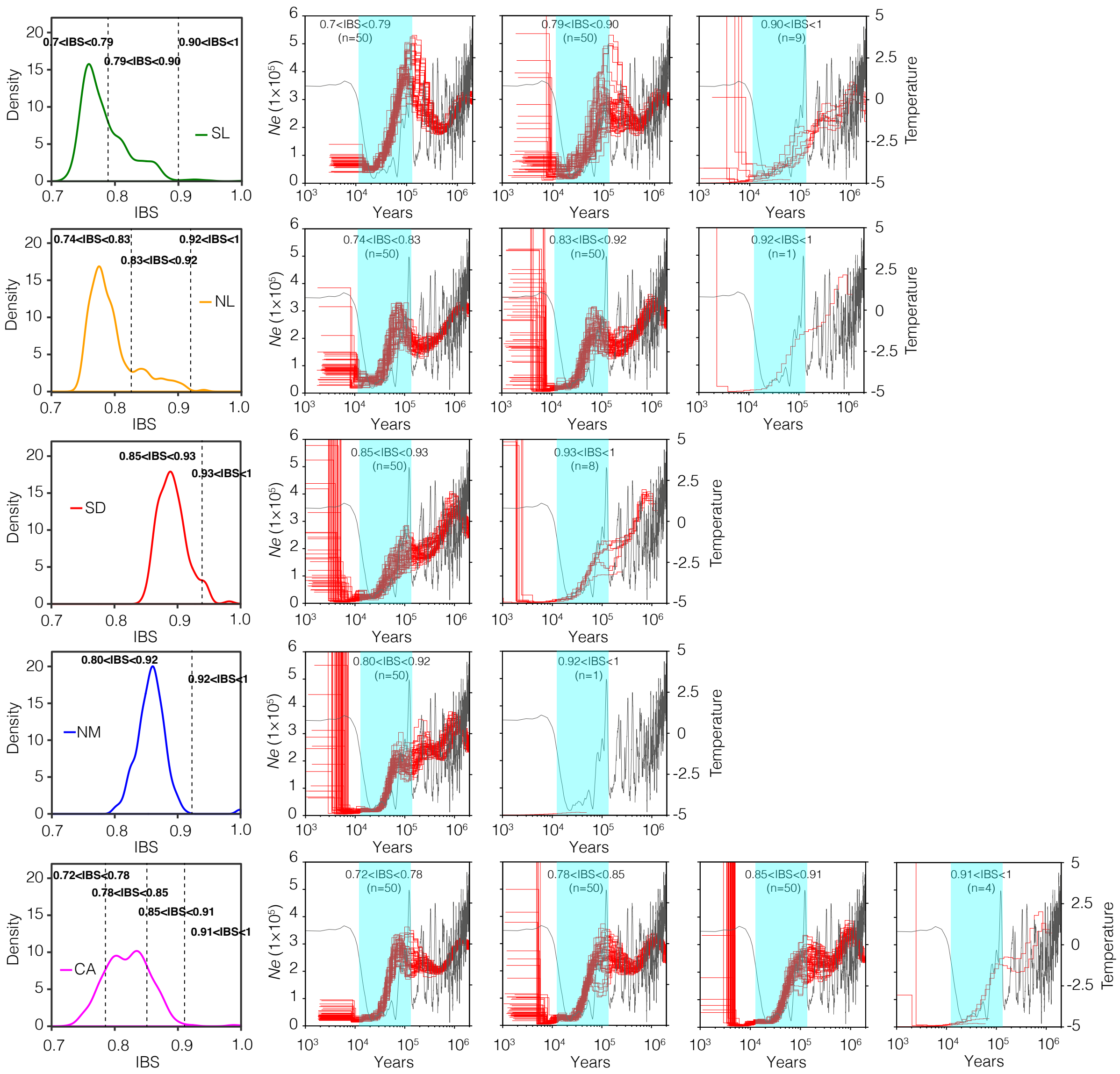

Supplementary Figure 2: Historic trajectories of effective population sizes in 5 wild barley populations inferred by PSMC. The left panel shows the density distribution of IBS values for all pairwise samples within each wild barley population. Based on the distribution, IBS values were divided into two to four bins. For each bin, 50 sample pairs were randomly selected (if the number of pairs exceeded 50), or all pairs were used (if the number of pairs was fewer than 50) to construct pseudo-diploid genome. The right panel shows the changes in effective population size over time inferred using PSMC. Each red line corresponds to one pseudo-diploid genomes. The dark gray line represents global temperature changes over the past 2 million years. The cyan-colored rectangle indicates the time span of the Last Glacial Period (LGP), from 120 ka BP to 11 ka BP.

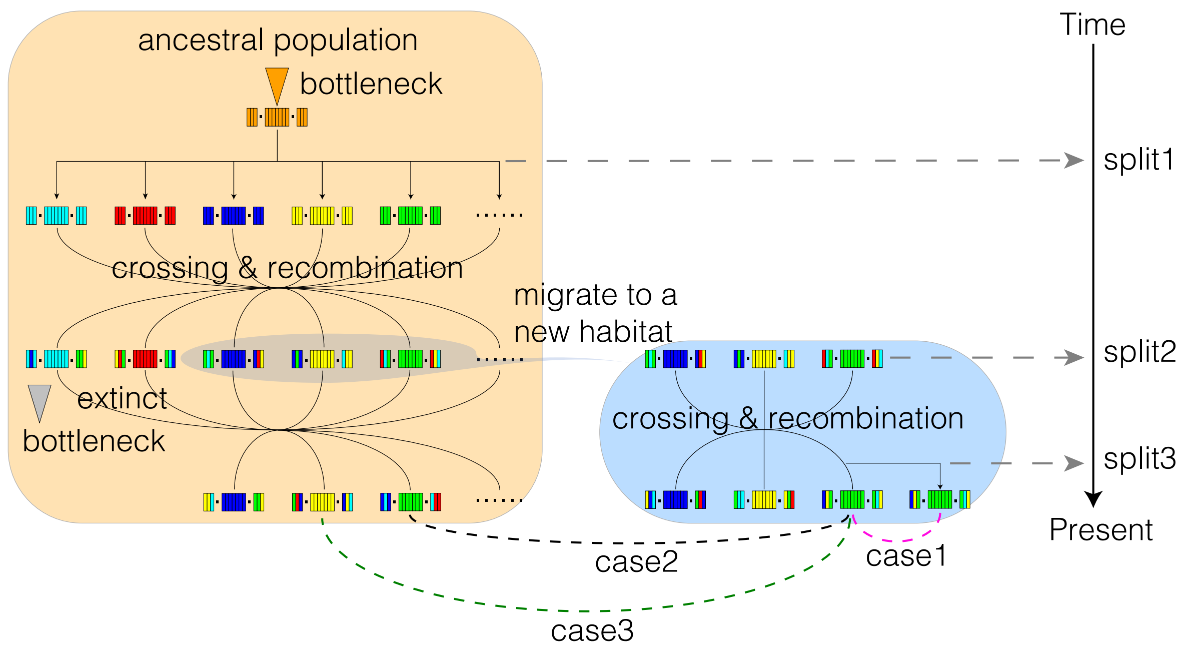

case1: the SNP number distribution is unimodal. Both the distal and proximal regions indicate split3.

case2: the SNP number distribution is bimodal. The distal region indicates split1, while the proximal region indicates split2.

case3: the SNP number distribution is unimodal. Both the distal and proximal regions indicate split1.

Supplementary Figure 3: Patterns of sequence divergence differ along the genome. This figure complements **Extended Data Fig. 2b.** The three numbered cases shown in both figures correspond to each other. The groups of colored bars each represent a single chromosome of different wild barley individuals. The outer bars stand for genomic windows in distal regions, the inner ones for those in proximal regions. After an initial bottleneck an ancestral population of wild barley split into structured subpopulations (represented by different colors). This process may have occurred independently in different mutually isolated populations (e.g. in the Southern Levant or Central Asia). Here, one local ancestral population is shown as an example. Gene flow between subpopulations reshuffled haplotypes over time (left-hand part). As the population colonized new habitats, bottlenecks occurred ancestral haplotypes were lost (right-hand part). Since the recombination rate in proximal regions is low, these can be considered as a single recombinational unit (~haplotype block). Because proximal regions are physically extensive, long shared haplotype blocks strongly influence the distribution of sequence divergence in windows of fixed physical size. In case1, two individuals are compared that come from the same extant wild barley population and neither has received recent gene flow from other populations. They share the same haplotype blocks in both distal and proximal regions and the distribution of sequence divergence is thus unimodal. In case2, two individuals come from a different population. Recombination and gene flow have reshuffled and broken up haplotypes in distal regions, whereas the proximal haplotypes trace back to a common ancestor at time point split2 and have remained intact in this scenario. Hence, the distribution of sequence divergence is bimodal: the peak in distal regions corresponds to early divergence (split1), whereas the peak in proximal regions reflects later divergence (split2) of the two lineages that lead to either individual. In case 3, also the proximal regions trace back to different ancestral haplotypes, which diverged early (split1). Hence, the distribution of sequence divergence along the genome is unimodal with a single early peak.

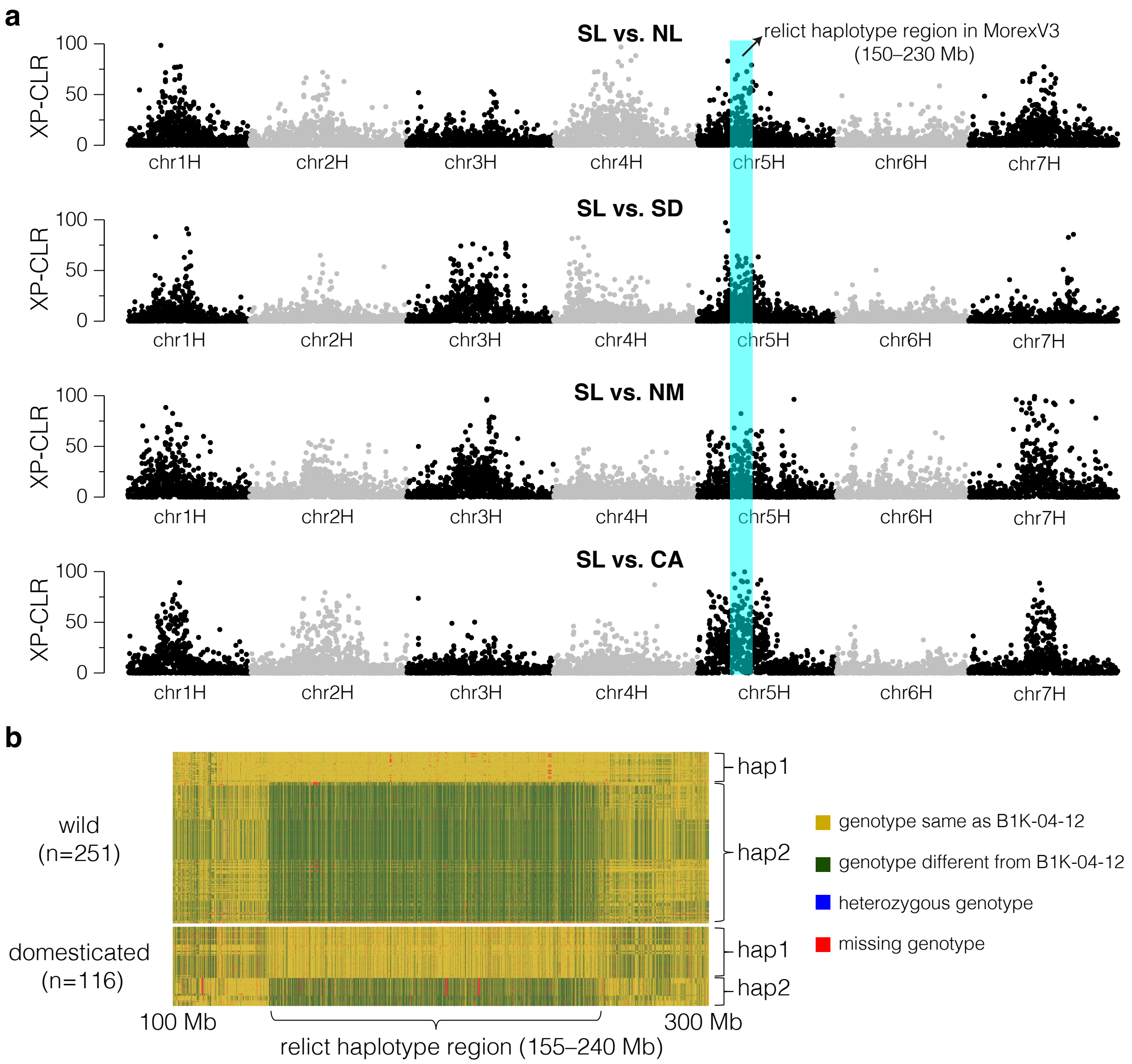

Supplementary Figure 4: Selective sweep in the relict haplotype region on chr5H. a. Selective sweep signals detected using XP-CLR. The SL group was compared against each of the other four wild barley populations separately. The analysis was performed with a window size of 1 Mb (shift: 200 kb). The cyan-colored rectangle marks the location of the relict haplotype region on chr5H in the MorexV3 reference genome (150–230 Mb). **b.** Haplotypes structure on chr5H in all high-coverage sequenced wild and domesticated barley accessions. The plot is based on an SNP matrix aligned to the reference genome of wild barley B1K-04-12 (Jayakodi et al. 2024). Hap1 and Hap2 were defined based on the analysis in **Extended Data Fig. 4b**.

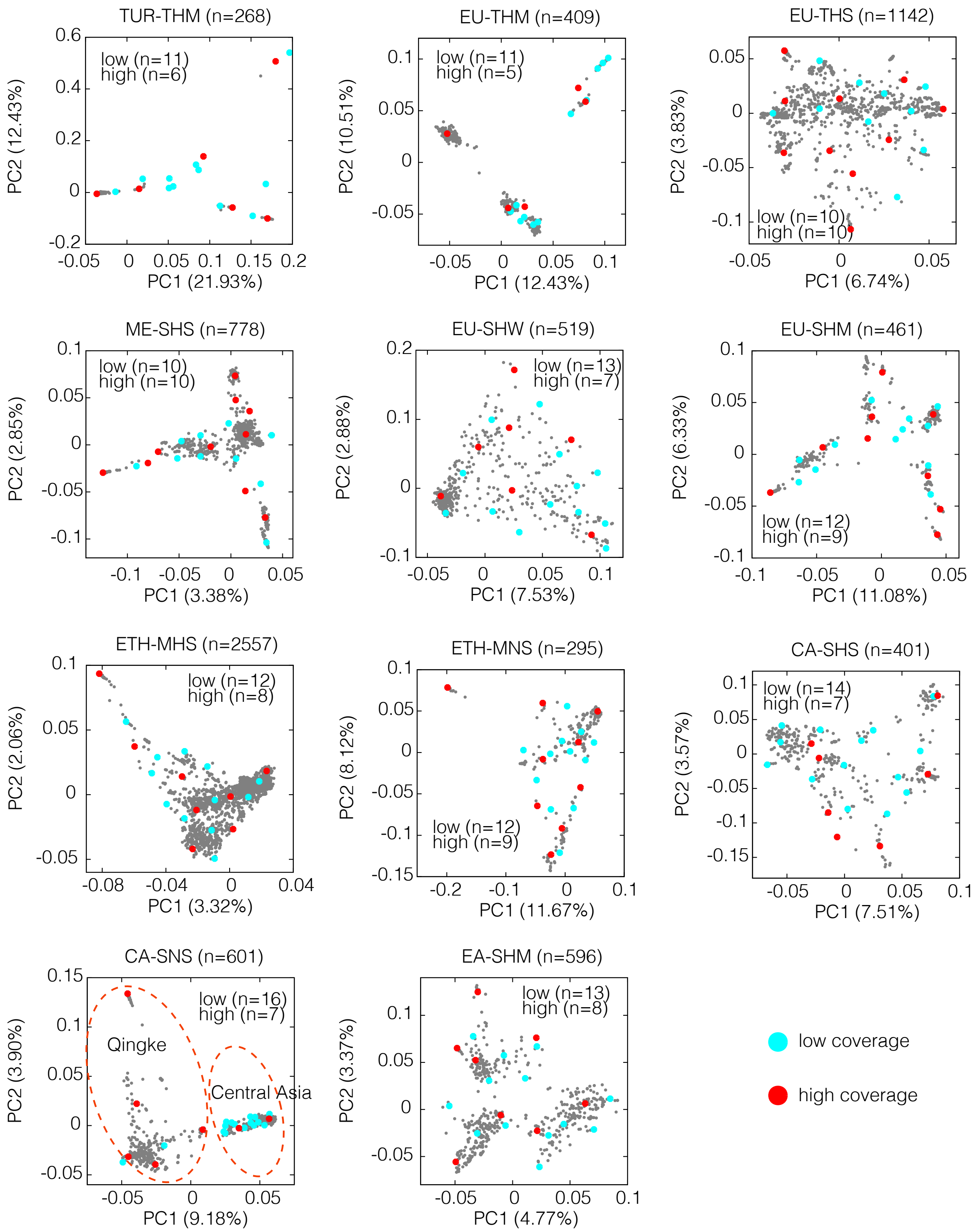

Supplementary Figure 5: Selection of samples for whole-genome sequencing from 11 domesticated barley populations. Milner et al. 2019 defined 12 populations of domesticated barley. The selection of samples from their "orange" population is shown in **Supplementary Fig. 6**. For each of the remaining 11 populations, PCAs were run for un-admixed samples, i.e. those with ADMIXTURE ancestry coefficients ≥ 0.95. Then, about 20 samples (13 high-coverage, 7 low-coverage) were selected to cover the PCA diversity space of each population. The left-hand cluster in the PCA of CA-SNS (Central Asia 6-rowed naked barleys) contains Qingke (Tibetan hulless barleys). This population was studied by Zeng et al.

2018 in detail and was not the focus of the present study. Hence, comparatively fewer Qingke samples were selected.

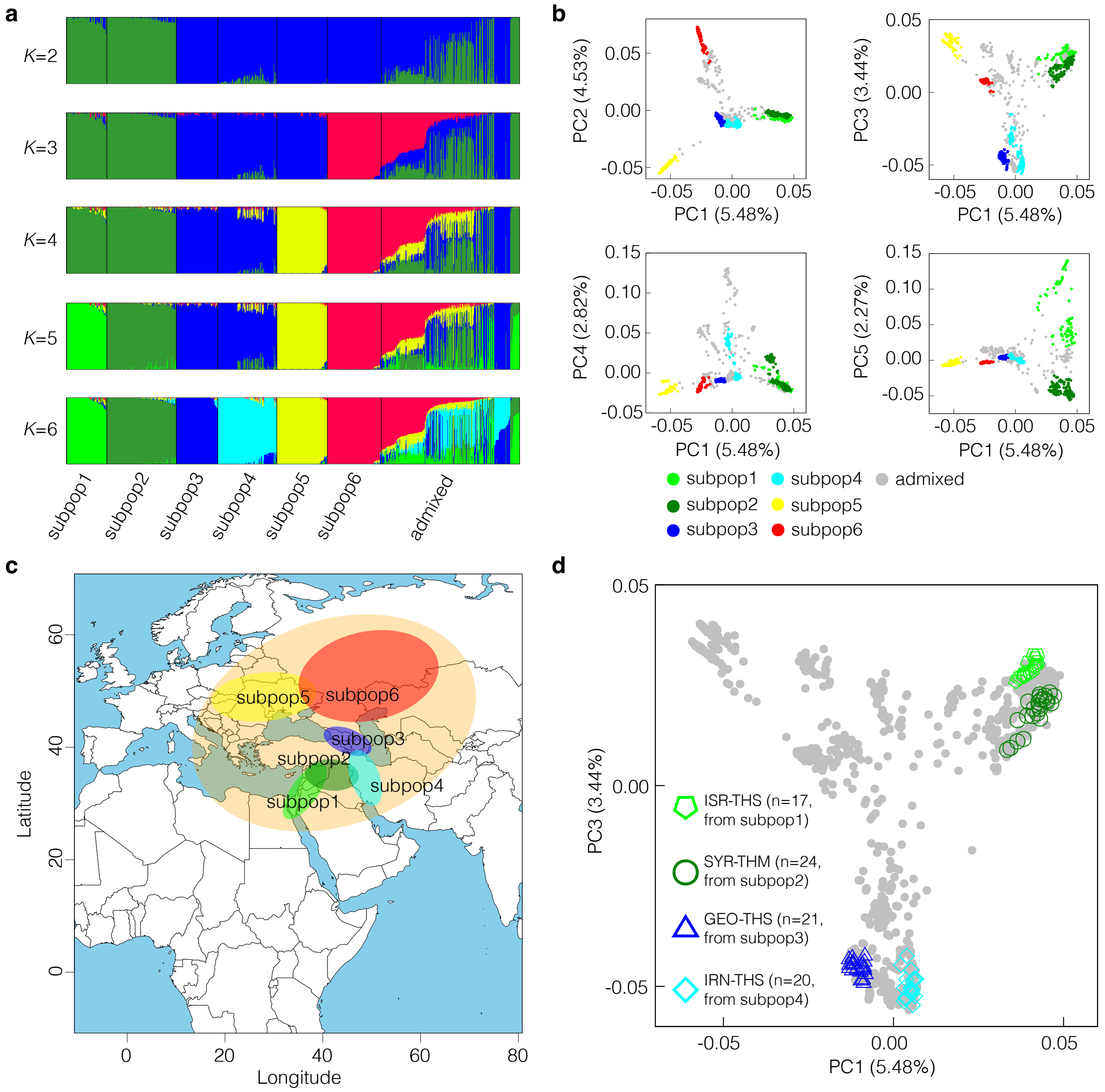

Supplementary Figure 6: Selection of samples for whole-genome sequencing from the "orange" population of Milner et al. 2019. This figure is a complement to the sample selection in **Supplementary Fig. 5**. **(a)** Individual ADMIXTURE ancestry coefficients with the number of ancestral populations (*K*) ranging from 2 to 6. ADMIXTURE was run on member of the Milner et al.'s "orange" population using their GBS data. Individuals whose major ancestry coefficient was less than 0.8 were considered admixed. **(b)** Diversity space of the "orange" population as revealed by PCA. The higher PCs (2 to 5) were plotted against PC1. Colors correspond to unadmixed samples as per panel **(a)**. **(c)** Predominant geographical origins of the subpopulations of "orange". Geographic outlines were obtained from the R package 'maps' (https://CRAN.R-project.org/package=maps), which uses public-domain base map data. **(d)** Samples from four populations in the Near East and Causasus that were selected for whole-genome sequencing are highlighted in the PCA.

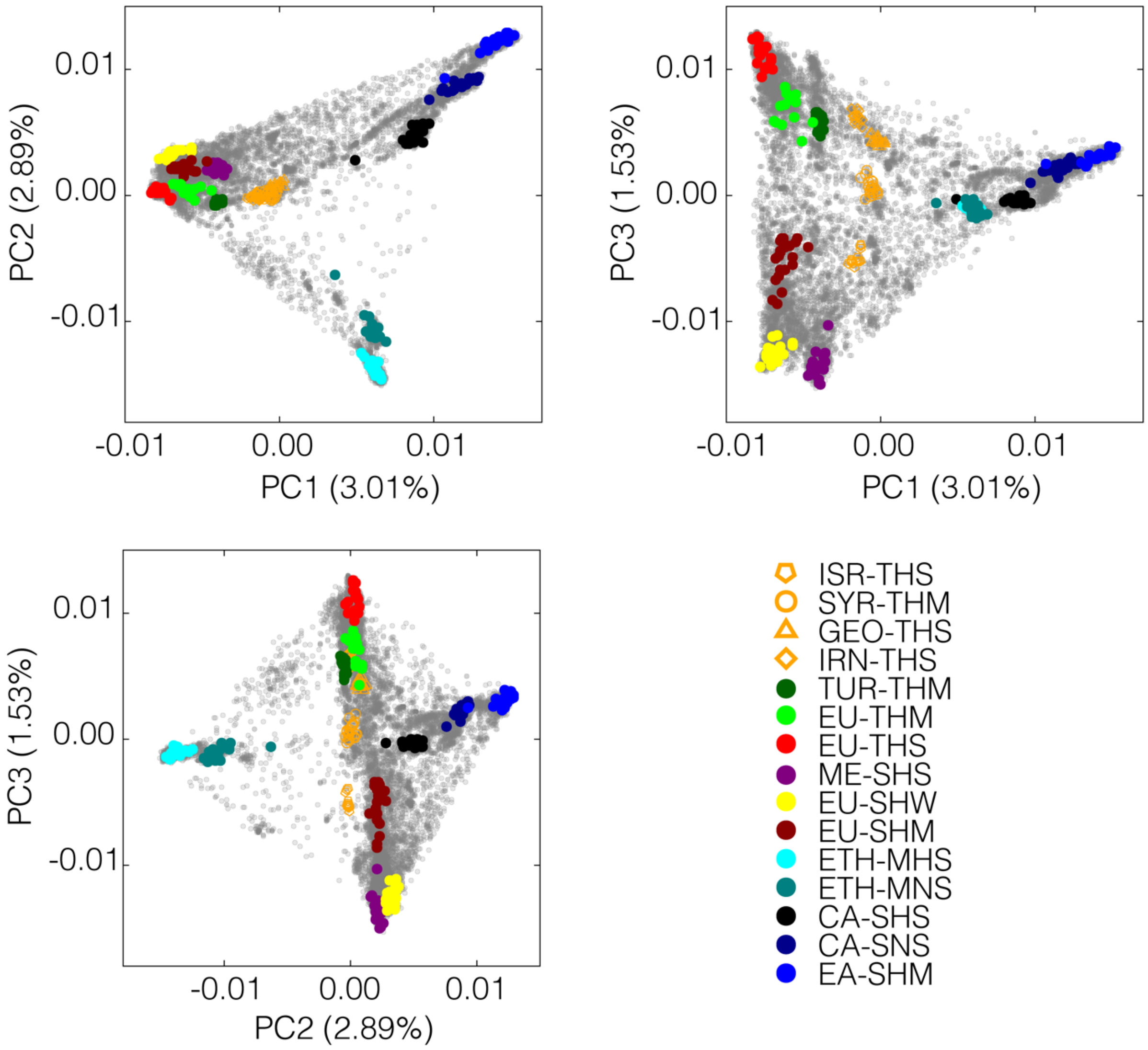

Supplementary Figure 7: Positions of 302 selected samples for whole-genome sequencing in the diversity space spanned by 19,778 domesticated samples of Milner et al. 2019.

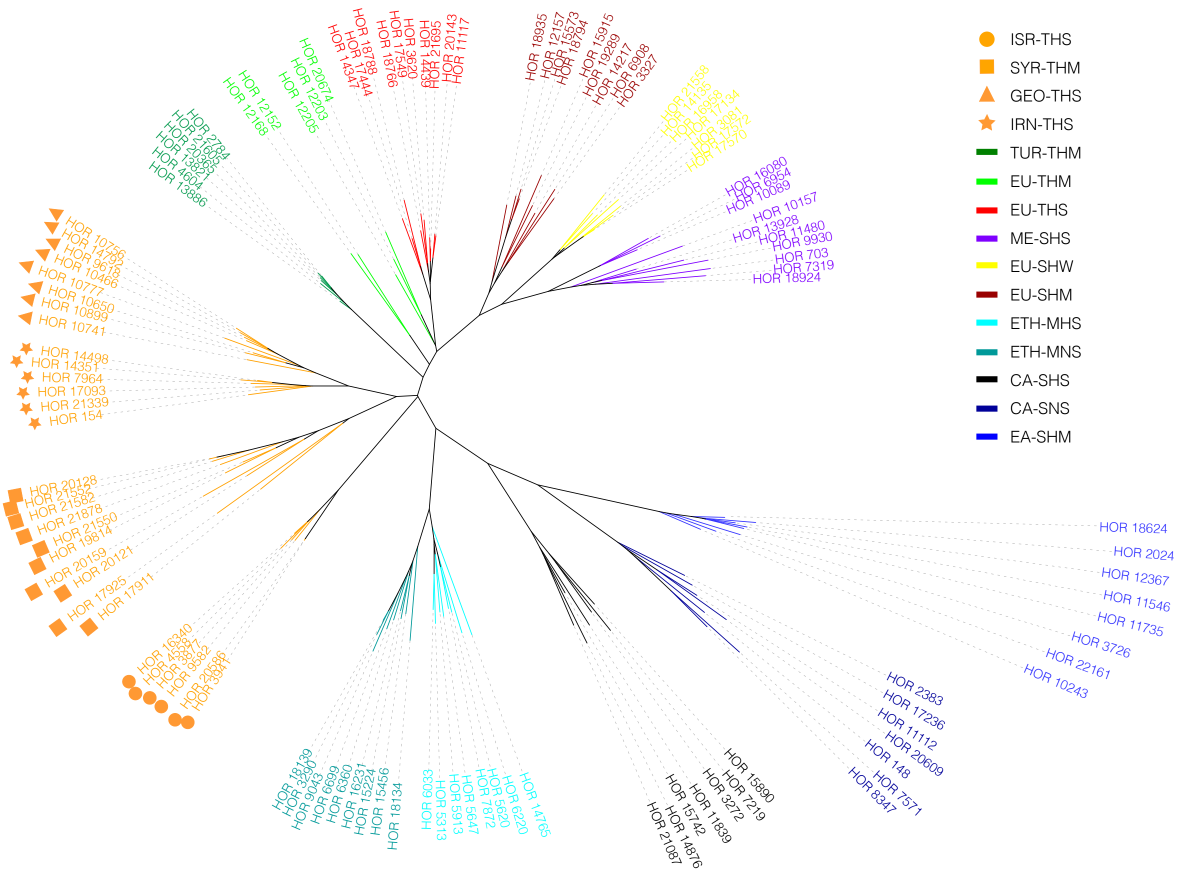

Supplementary Figure 8: Unrooted neighbor-joining phylogenetic tree constructed from 116 domesticated barley accessions based on high-coverage whole-genome sequencing data.

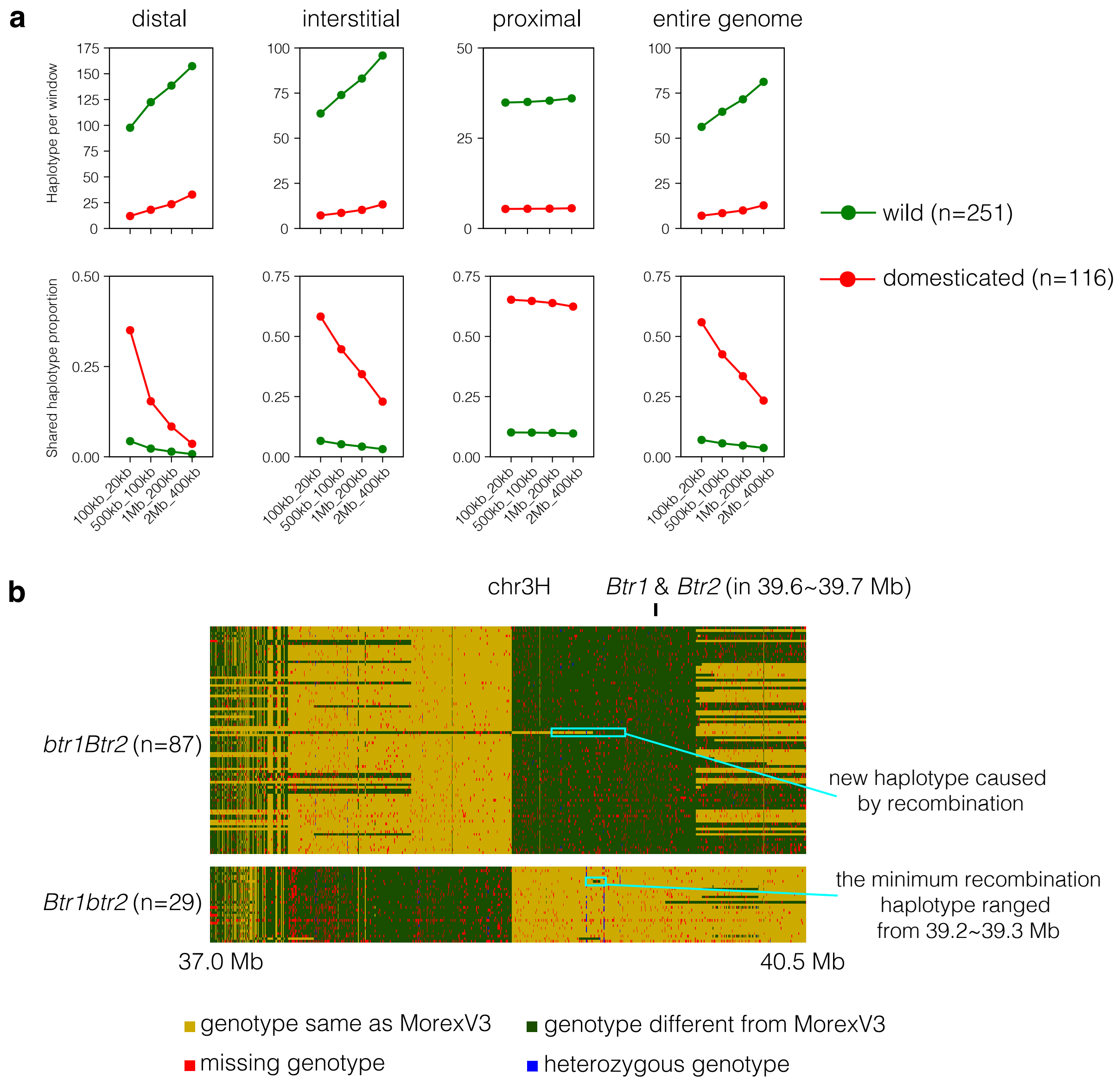

Supplementary Figure 9: Effect of window size on haplotype definition. (a) Haplotype numbers and proportions of shared haplotypes in wild and domesticated barleys were computed from IntroBlocker runs with different window sizes: 100 kb (shift: 20 kb), 500 kb (shift: 100 kb), 1 Mb (shift: 200 kb), 2 Mb (shift: 400 kb). As the window size increases, so does the number of haplotypes, whereas the proportion of shared haplotypes decreases. The pattern is less pronounced in proximal, recombination-poor regions where haplotypes are longer. **(b)** The haplotype structure at the *btr1/2* locus is shown as an example. The samples shown are cultivated barleys with recombinant haplotypes. If the window size is large, more recombinant haplotypes are considered novel. The length of the smallest sequence exchange we observed between the original haplotypes (*Btr1btr2* and *btr1Btr2*) was 100 kb. Hence we used this window size (with a 20 kb shift) to compile the haplotype matrix for subsequent analyses.

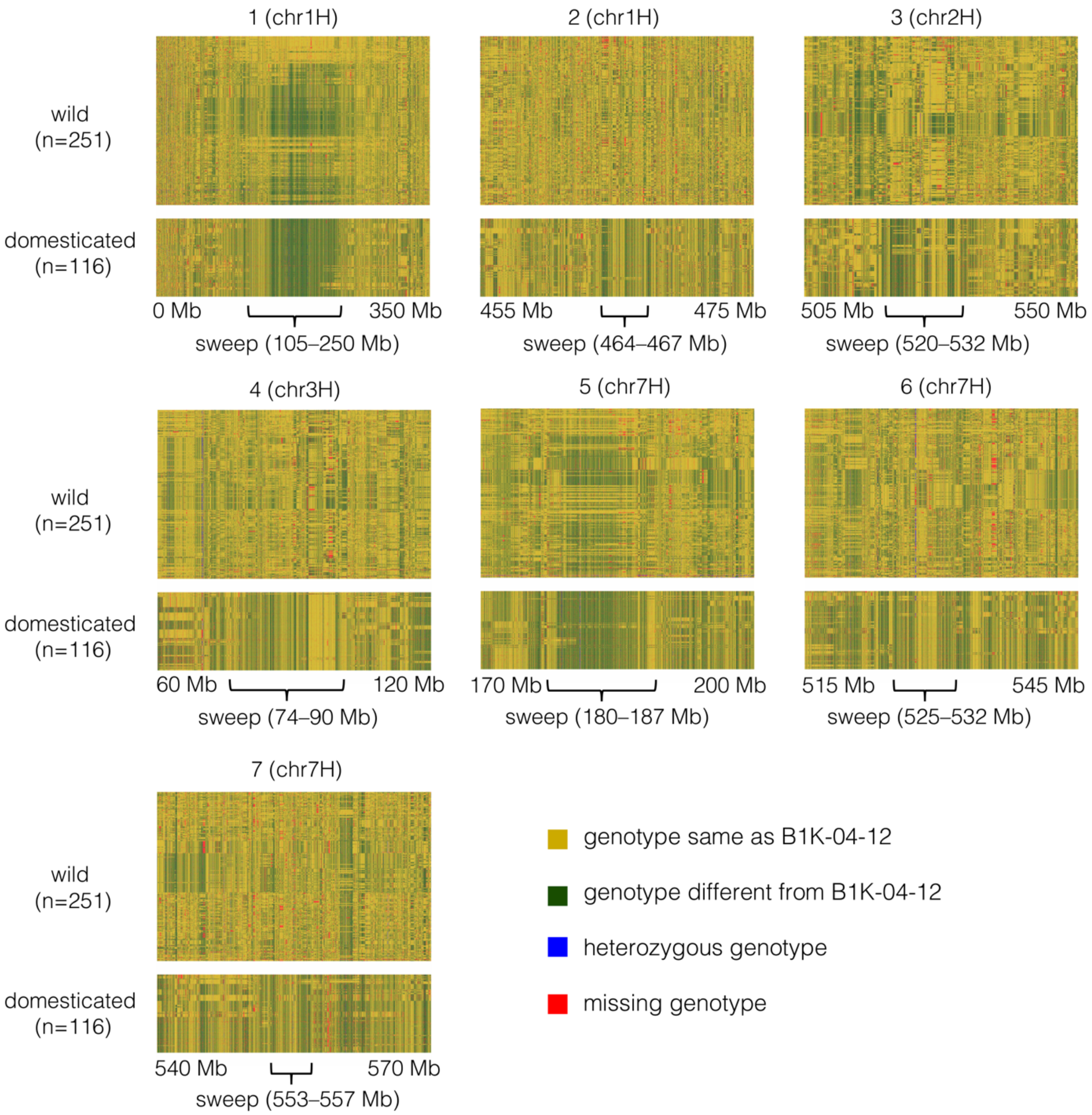

Supplementary Figure 10: SNP haplotypes in the seven selective sweep regions shown in Fig. 3g. The reference for SNP matrix was the wild barley B1K-04-12 from the barley pangenome (Jayakodi et al. 2024). Genotype calls were color-coded as indicated. Only SNPs sites with fewer than 20% missing calls and a minor allele frequency above 10 % were used.

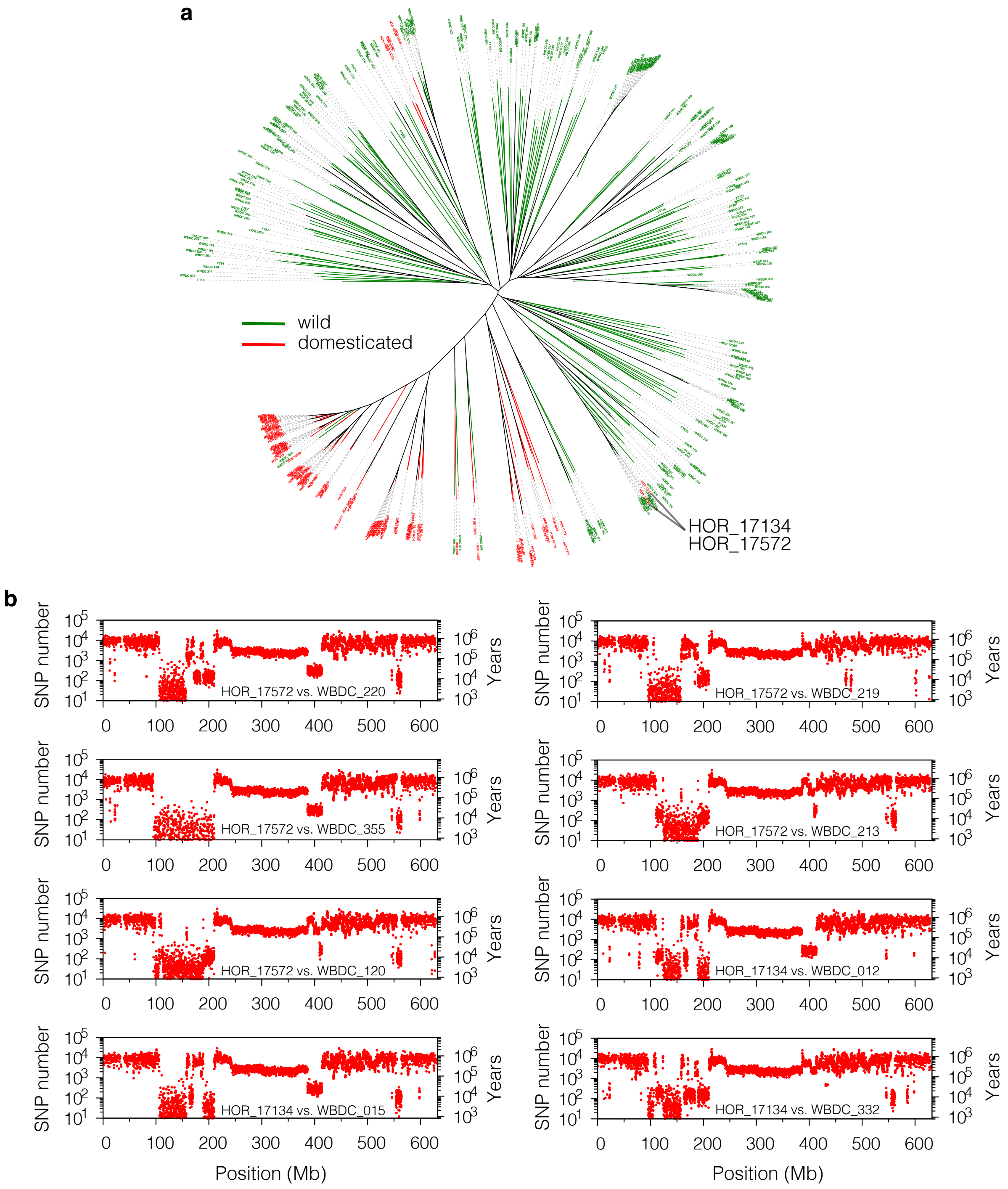

Supplementary Figure 11: Recent gene flow from Eastern Asia wild barley to European 6-rowed winter barleys on chromosome 7H. (a) Neighbor-joining tree computed from 1.85 M biallelic SNPs in the interval 120 Mb to 160 Mb on chromosome 7H. Two accessions, HOR 17134 and HOR 17572, cluster with wild barleys. **(b)** Sequence divergence (SNPs per Mb) between these two accessions and different wild barleys in 100 kb windows (shift: 20 kb).

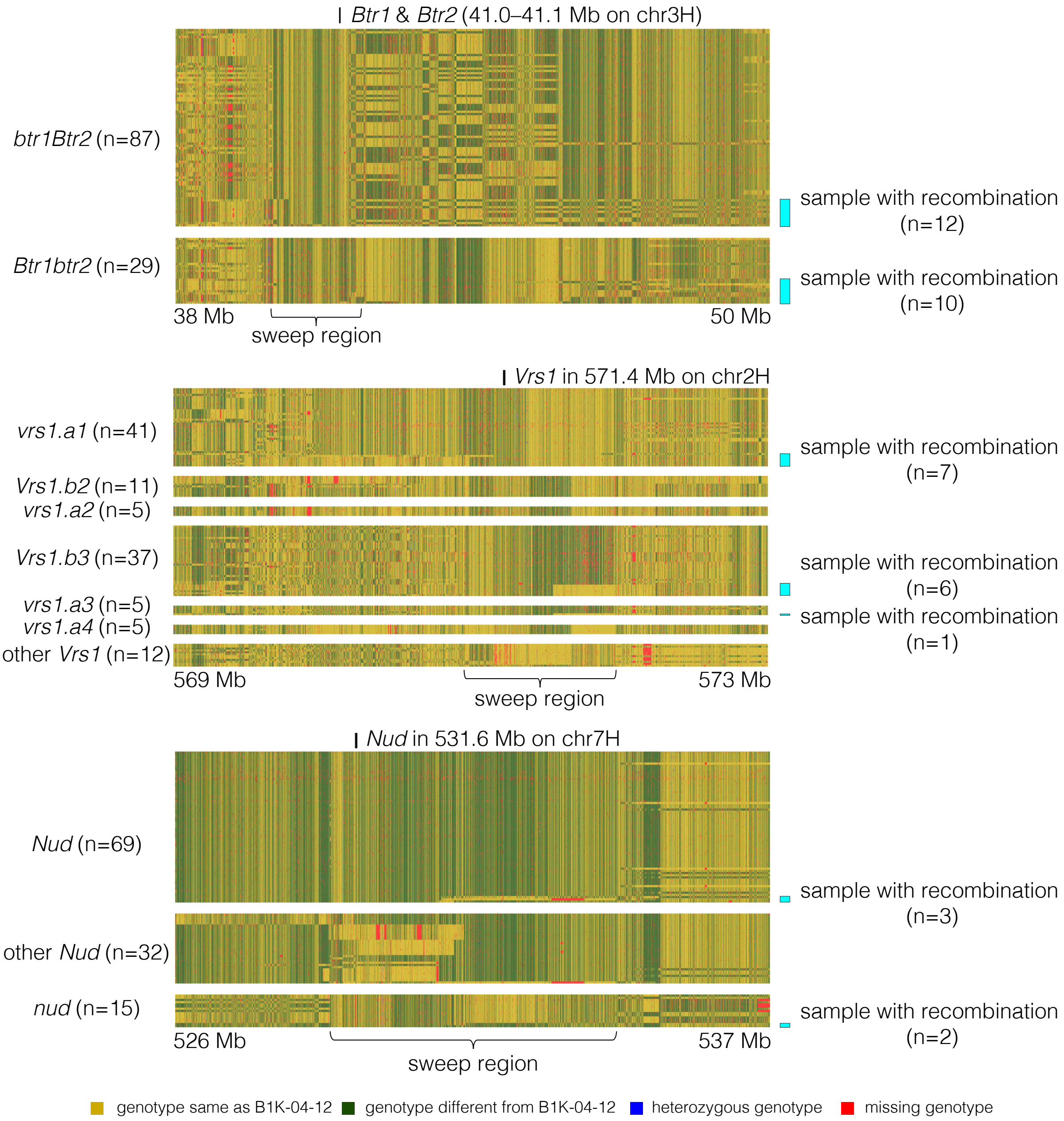

Supplementary Figure 12: Haplotype structure of 116 high-coverage domesticated barley surrounding the *Btr1/2*, *Vrs1* and *Nud* gene loci. The reference for SNP matrix was the wild barley B1K-04-12 from the barley pangenome (Jayakodi et al. 2024). Only SNP sites with MAF ≥ 0.1 were used for plotting. The cyan-colored rectangles indicate samples carrying recombinant haplotypes in the sweep region. These samples are excluded from the SNP age estimation. Samples with "other *Vrs1*" and "other *Nud*" haplotype were also not used for SNP age estimation.

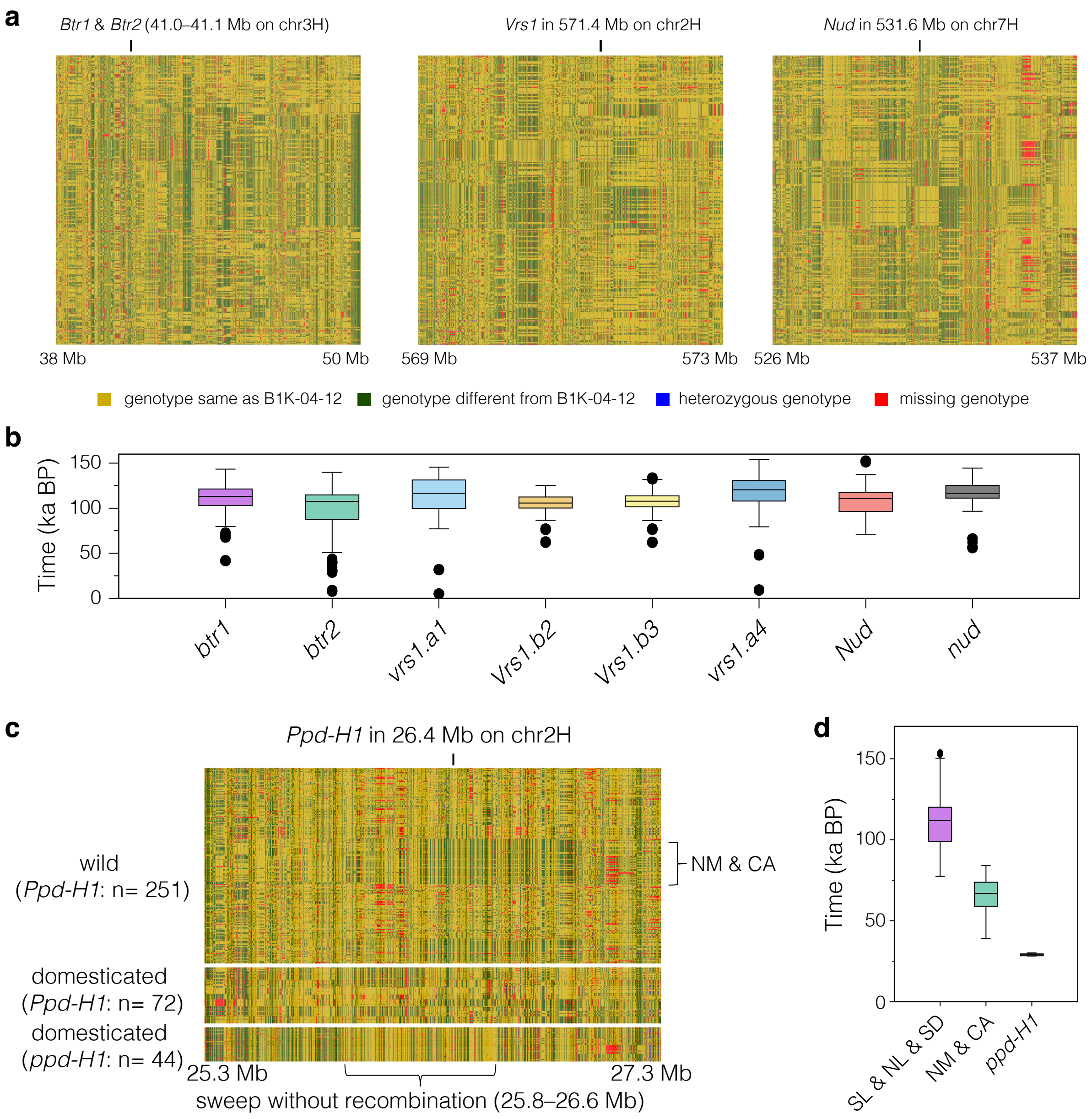

Supplementary Figure 13: SNP ages of control group. (a) Haplotype structure of 251 wild barley accessions surrounding the *Btr1/2*, *Vrs1* and *Nud* gene loci. (**b**) Estimated ages of SNPs (0.2 < allele frequency < 0.5) derived from wild barley, calculated using GEVA. To clarify the meaning of the x-axis labels, take "*btr1*" as an example: when estimating the age of the domesticated *btr1* allele, we selected approximately 40 haplotype-specific private SNPs located between 41.08 Mb and 41.18 Mb. As a wild control group, we selected another ~40 SNPs from wild barley that are located within the same genomic interval. Therefore, the label "*btr1*" here refers to the estimated ages of wild barley SNPs located in the same genomic region used for calculating the age of the domesticated *btr1* haplotype. Each box plot is based on n = S × 10, where S represents the number of SNP sites used, and the data were generated by repeating the analysis 10 times with different random seeds. Specifically, for each allele, the number of data points used to generate the box plots is as follows: *btr1* (n = 42 × 10), *btr2* (n = 41 × 10), *vrs1.a1* (n = 40 × 10), *Vrs1.b2* (n = 31 × 10), *Vrs1.b3* (n = 50 × 10), *vrs1.a4* (n = 45 × 10), *Nud* (n = 42 × 10), and *nud* (n = 45 × 10). **(c)** Haplotype structure of wild and domesticated barley around the *Ppd-H1* locus. **(d)** Estimated ages of SNPs

associated with different *Ppd-H1* haplotypes, calculated using GEVA. "SL & NL & SD": SNPs frequently found in the SL, NL, and SD populations; "NM & CA": SNPs frequently found in the NM and CA populations; "*ppd-H1*": causal SNP of recessive domesticated *ppd-H1* haplotype. Each box plot is based on n = S × 10, where S represents the number of SNP sites used, and the data were generated by repeating the analysis 10 times with different random seeds. Specifically, for each allele, the number of data points used to generate the box plots is as follows: SL & NL & SD (n = 43 × 10), NM & CA (n = 47 × 10), *ppd-H1* (n = 1 × 10). Details of SNPs selection from control group can be found in **Supplementary Table 15**. In panels **(b)** and **(d)**, box plots display the median (center line), the 25th and 75th percentiles (box bounds), and whiskers that extend to values within 1.5× the interquartile range (IQR); outliers beyond this range are shown as individual points.

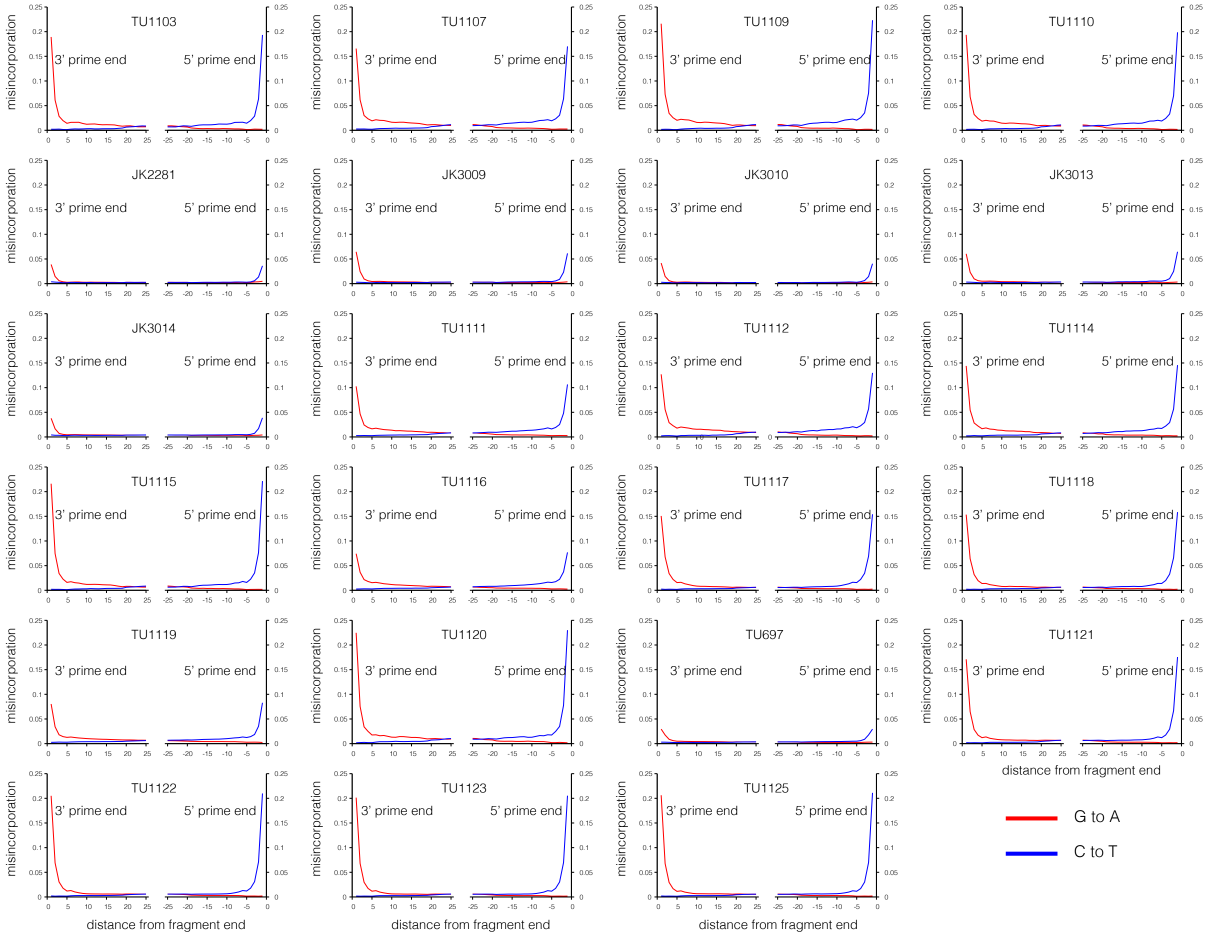

Supplementary Figure 14: Nucleotide misincorporation profiles in the sequence data 23 ancient DNA samples.

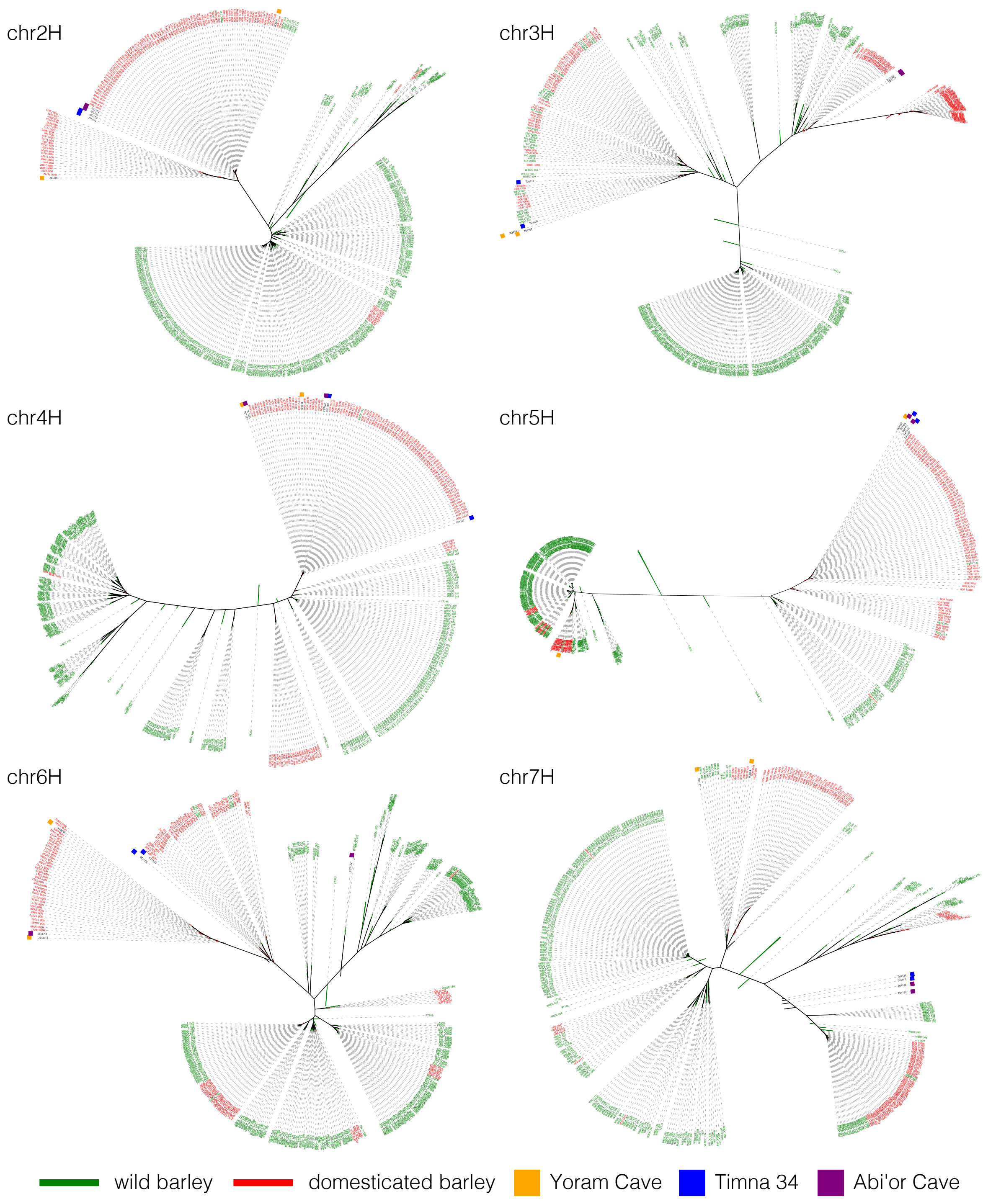

Supplementary Figure 15: Phylogenetic tree illustrating haplotypes around the pericentromeric region. High-coverage sequencing data were used from wild barley (n = 251), domesticated barley (n = 116), and ancient barley (n = 6). SNPs located within ±25 Mb of the centromere were used for tree construction.

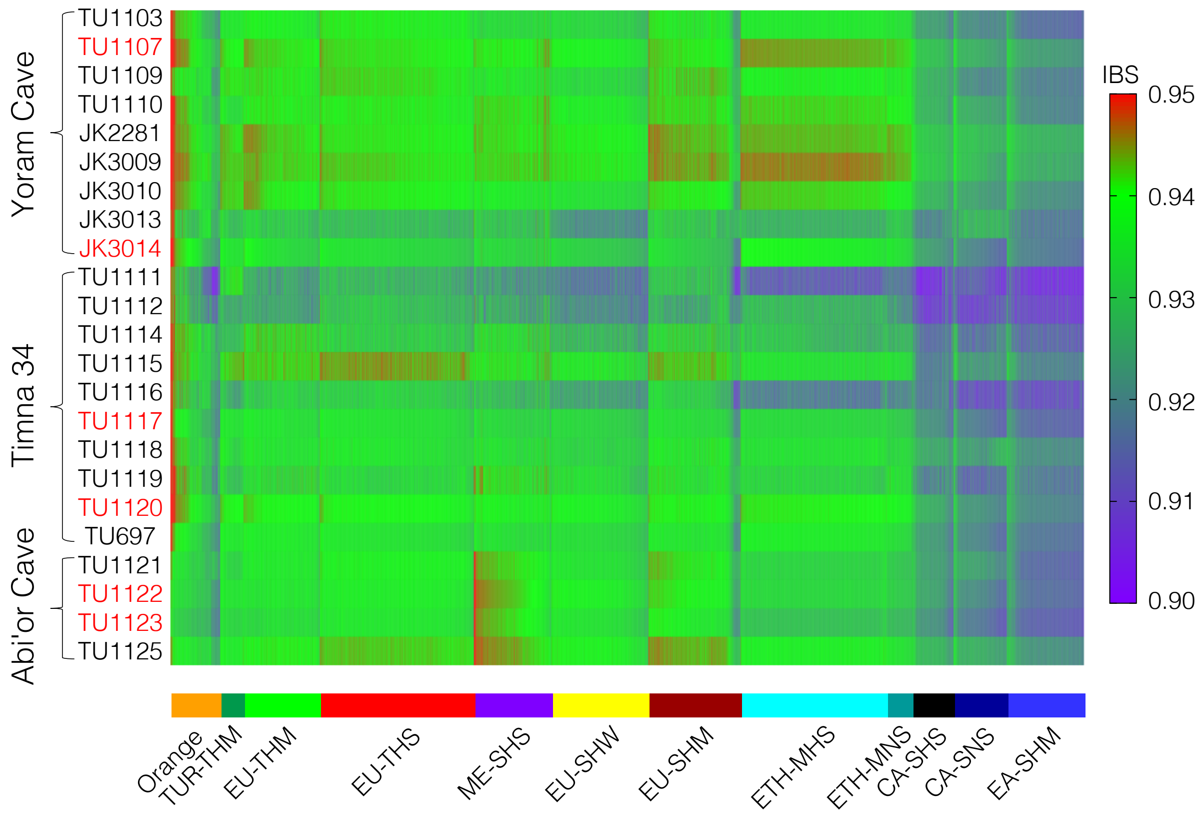

Supplementary Figure 16: Heatmap showing the identity-by-state (IBS) similarity between 23 ancient samples and 19,778 domesticated samples genotyped by Milner et al. 2019. Red font marks ancient samples sequenced at high coverage. Ancient samples with high similar to the "Orange" and ME-SHS population. The "Orange" population included Western Asian samples.

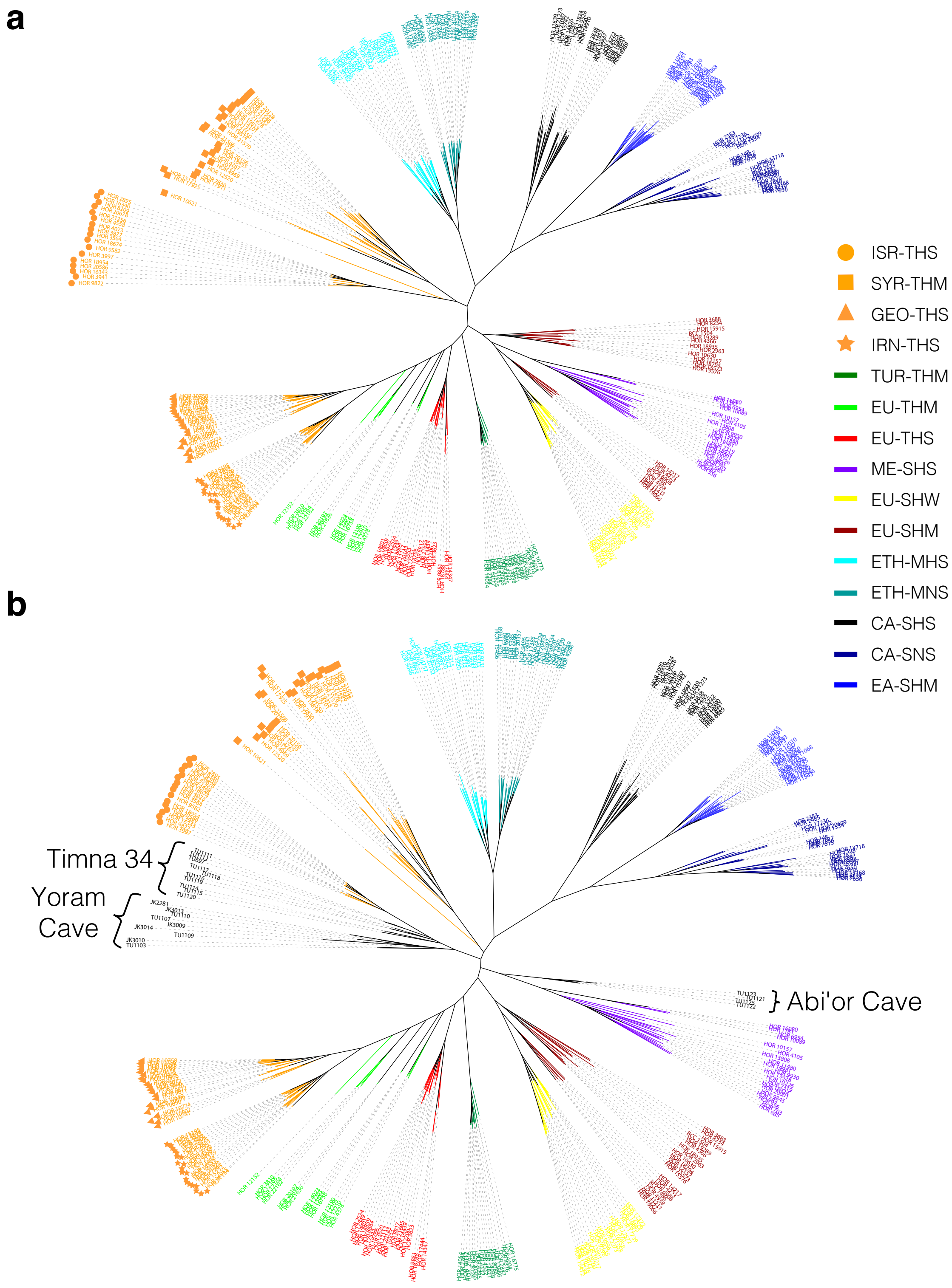

Supplementary Figure 17: Phylogenetic tree showing the relationships between 23 ancient and 302 modern barley accessions. (a) Unrooted neighbor-joining phylogenetic tree constructed only from 302 modern domesticated barley accessions. **(b)** Unrooted neighbor-joining phylogenetic tree constructed from 23 ancient and 302 modern domesticated barley accessions. SNP sites involving C→T and G→A transitions were not used in panel **(b)**.

missing rate ≤ 0.2

	ancient individual	modern individual	wild barley population				
SNP site			s1	s2	s3	s4	……
SNP1	G	A	A	A	G	A	A
SNP2	A	A	G	A	A	-	A
SNP3	A	G	A	G	A	A	A
SNP4	A	A	A	A	A	A	G
SNP5	G	A	A	G	A	A	A
shared rare allele	2	1					

neither individual has missing data

Supplementary Figure 18: The method used to compare genetic diversity between modern and ancient barley at the single-sample level. Red "G" indicates a rare allele found in wild barley. A '–' denotes missing genotype data in wild barley. Suppose M is the number of rare wild alleles shared by a modern barley sample, and A is the number shared by an ancient barley sample. We define the relative diversity change as:

Relative diversity change = (M - A) / A

A positive value indicates an increase in diversity in modern barley relative to ancient barley, while a negative value indicates a decrease. In the example shown, Relative diversity change = (1 - 2) / 2 = -0.5, meaning that modern barley has experienced a 50% reduction in diversity compared to ancient barley.

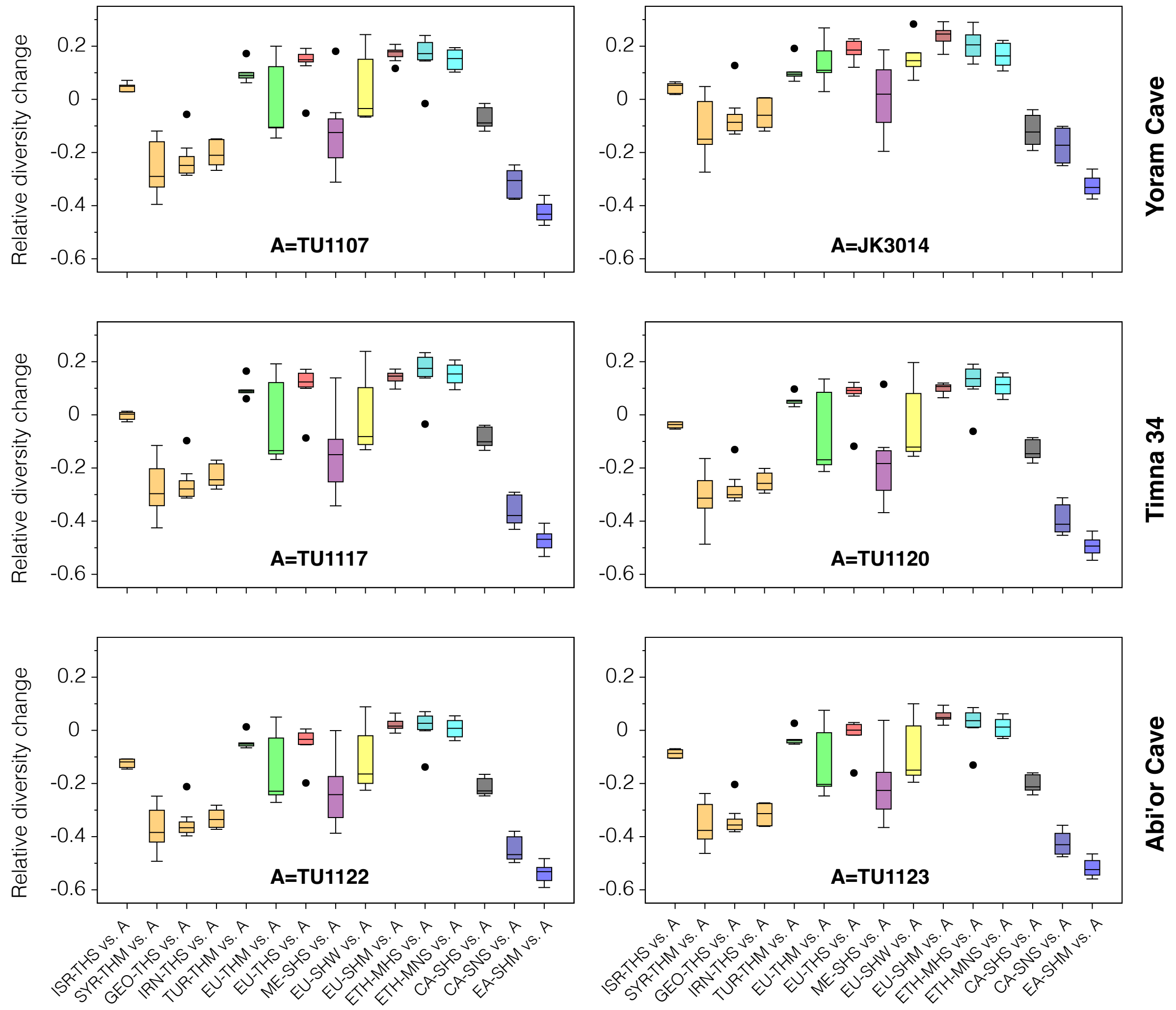

Supplementary Figure 19: Comparison of genetic diversity between samples from 15 modern barley populations and six high-coverage ancient barley individuals at the single-sample level. The definition of relative diversity change follows **Supplementary Fig. 18**. X-axis labels indicate pairwise comparisons in the format Modern population vs. A (ancient sample). SNP sites with rare variants in wild barley (0 < rare allele frequency ≤ 0.01) were used. Each box plot is based on n biologically independent sample pairs (Modern vs. ancient), each consisting of one modern and one ancient barley accession. The number of sample pairs for each group of modern and ancient barley is as follows: ISR-THS (n=6), SYR-THM (n=10), GEO-THS (n=8), IRN-THS (n=6), TUR-THM (n=6), EU-THM (n=5), EU-THS (n=10), ME-SHS (n=10), EU-SHW (n=7), EU-SHM (n=9), ETH-MHS (n=8), ETH-MNS (n=9), CA-SHS (n=7), CA-SNS (n=7), EA-SHM (n=8). Box plots show the median (center line), the 25th and 75th percentiles (box bounds), and whiskers extend to values within 1.5× the interquartile range (IQR); outliers beyond this range are shown as individual points.

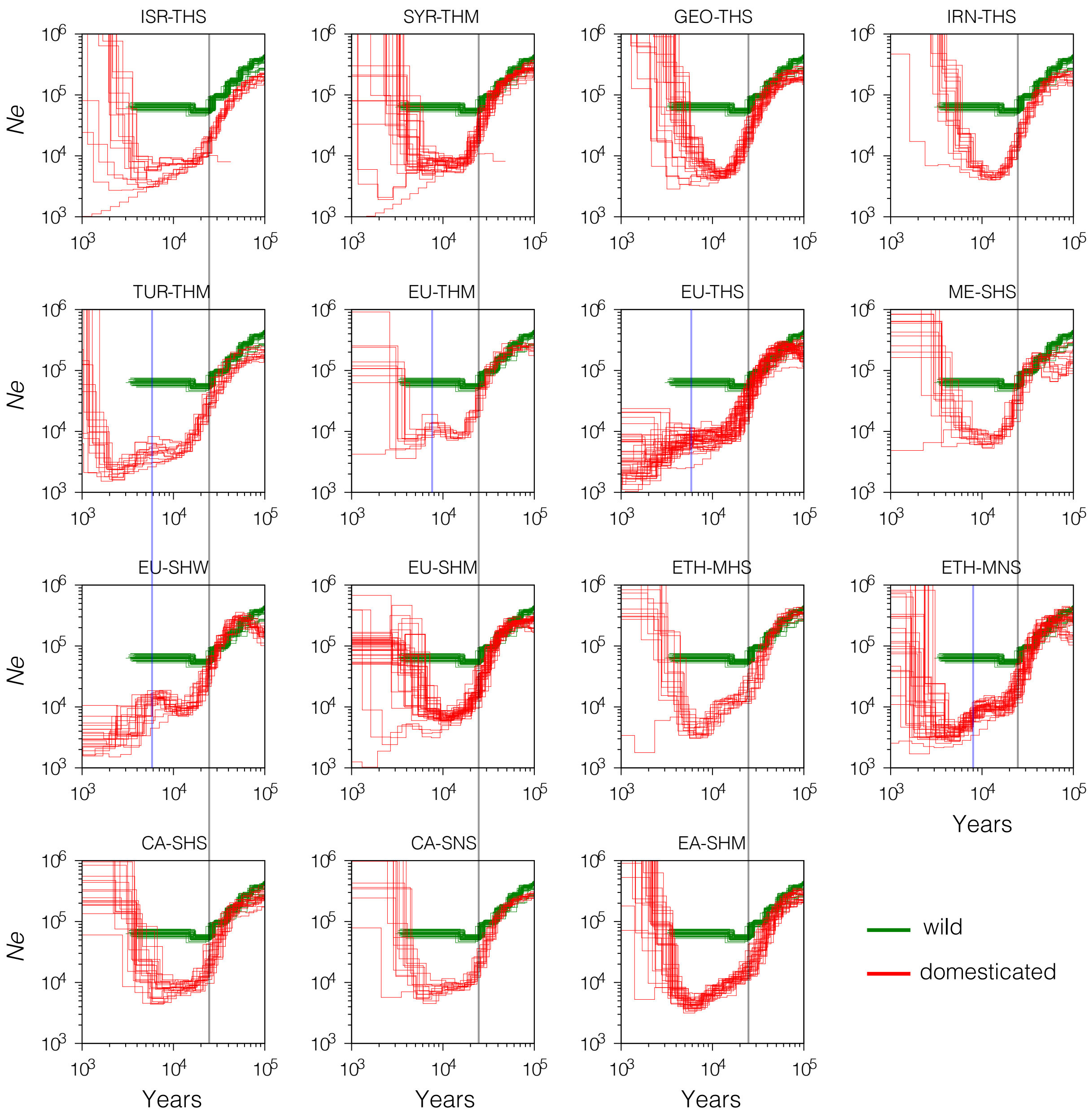

Supplementary Figure 20: Historic trajectories of effective population sizes in wild and domesticated barley as inferred by PSMC. Each red line stands for one of pseudo-diploid genomes representing all pairwise combination of samples in the respective domesticated populations. The green lines, shared across all panels, represent pseudo-diploid genomes derived from wild barley sample pairs listed in **Supplementary Table 23**. To capture the average demographic history of wild barley, we used only PSMC results from sample pairs with the lowest IBS range of 0.60–0.67 (**Extended Data Fig. 3b**). The grey line indicates the onset of divergence between wild and domesticated populations, beginning approximately 25 ka BP. In some domesticated barley populations, a blue line denotes a subsequent bottleneck event associated with geographic range expansion.

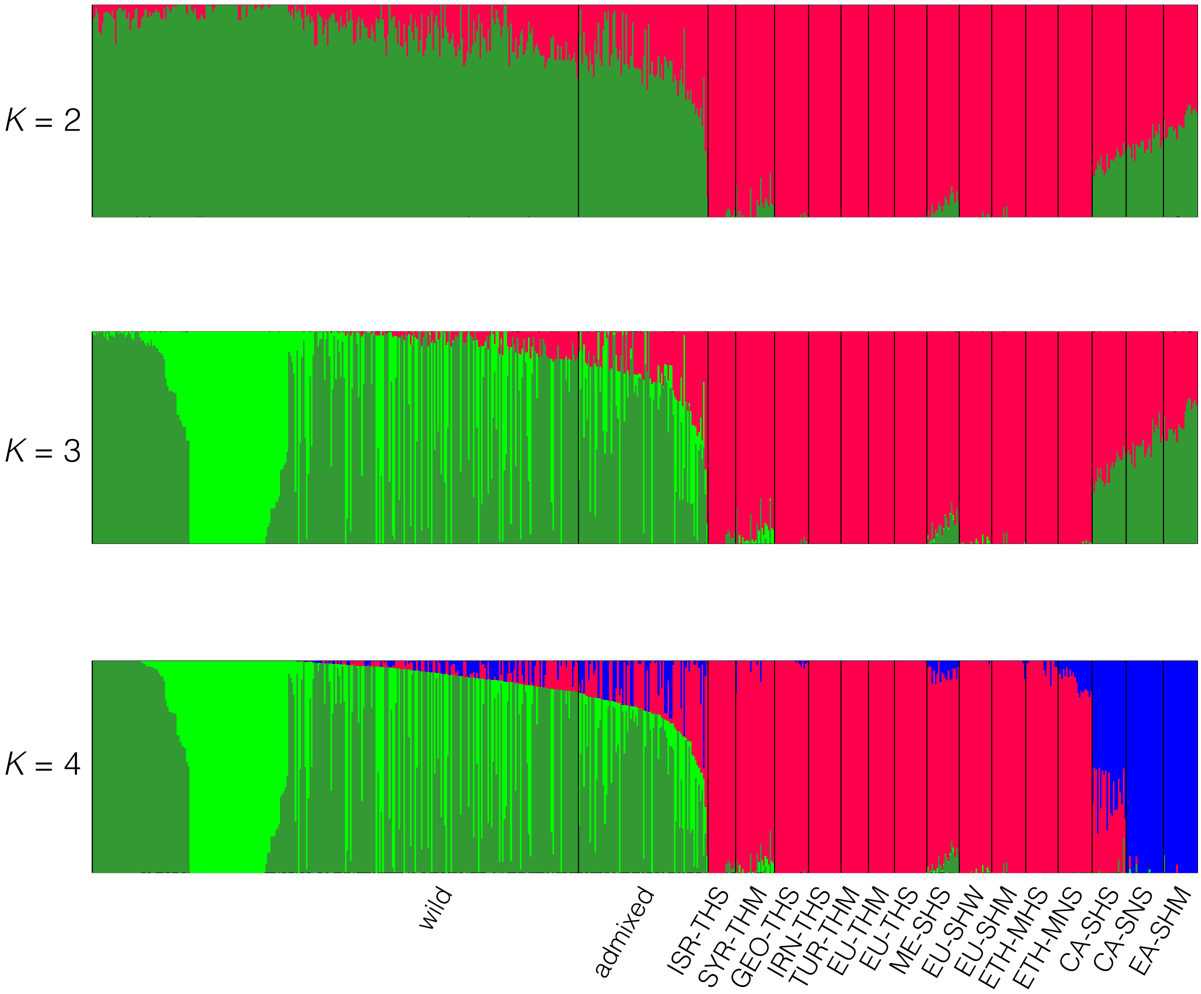

Supplementary Figure 21: Ancestry coefficients for 682 barley samples, as inferred using ADMIXTURE with the number of ancestral populations (*K*) ranging from 2 to 4. Individuals labeled as "wild" in the passport data but exhibiting less than 85% assignment to the "wild" ancestry component (shown in green) were considered admixed and excluded from downstream analyses.

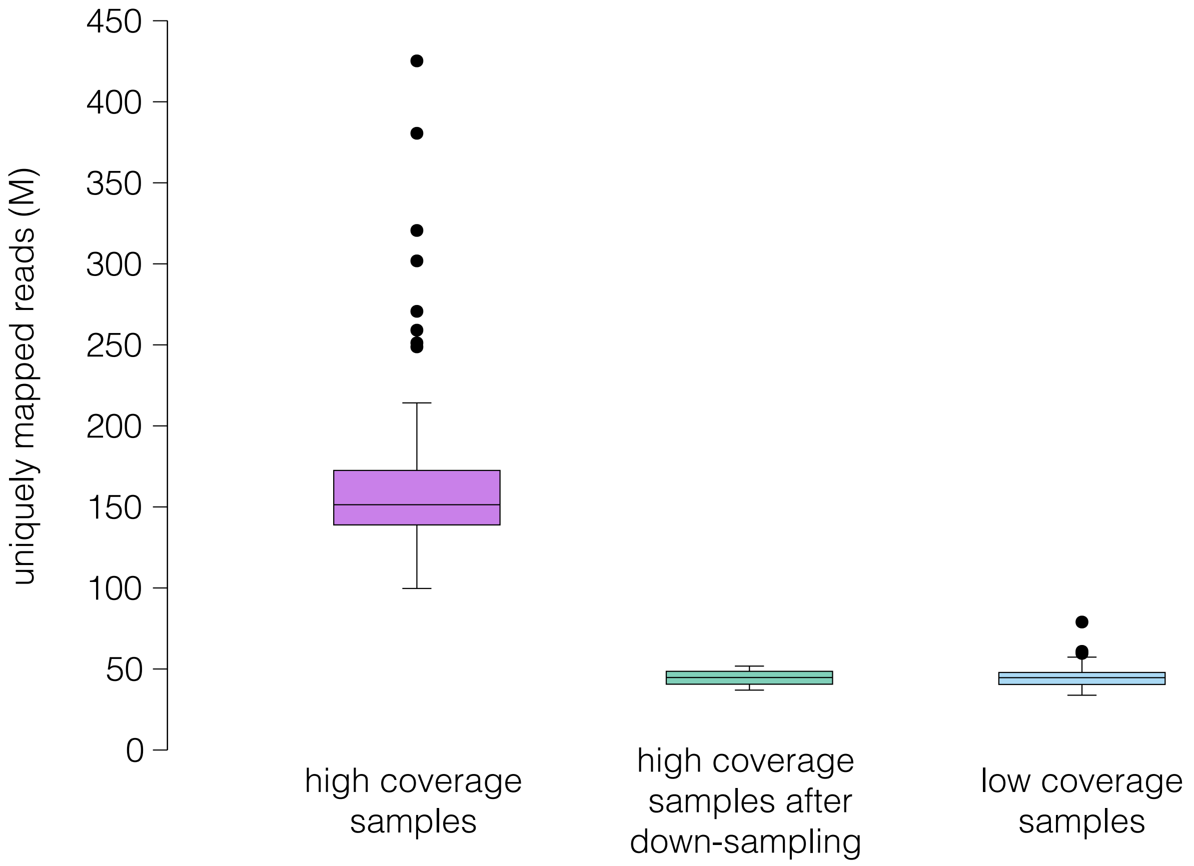

Supplementary Figure 22: Down-sampling of high-coverage sequence data. Box plots showing the distribution of the numbers of uniquely mapped reads (MAPQ ≥ 20) for 116 high-coverage and 186 low-coverage samples. For some analyses, high-coverage data were down-sampled to the level of the low-coverage samples. Box plots show the median (center line), the 25th and 75th percentiles (box bounds), and whiskers extend to values within 1.5× the interquartile range (IQR); outliers beyond this range are shown as individual points.

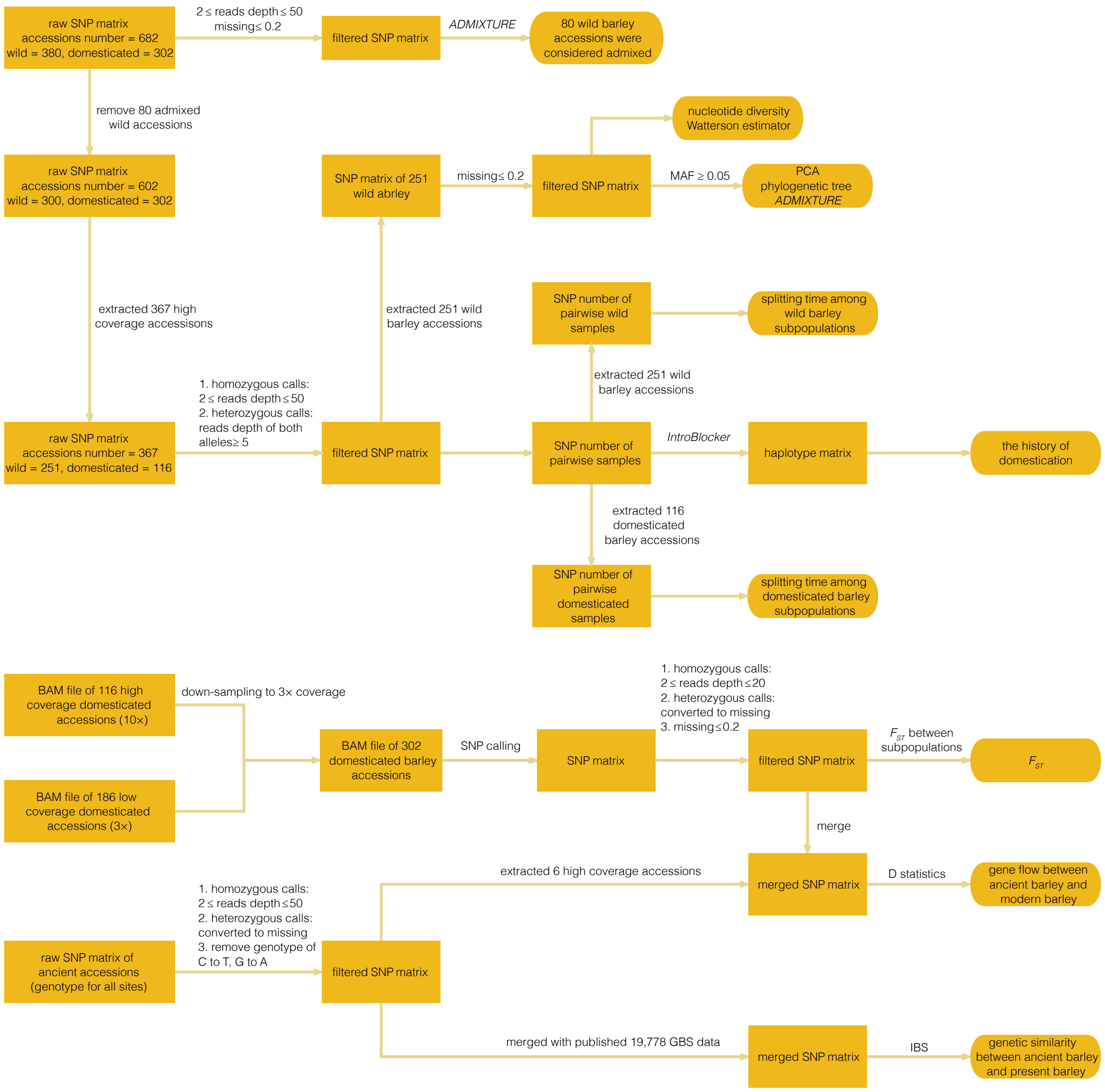

Supplementary Figure 23: Workflow of analyses conducted on SNP matrices.

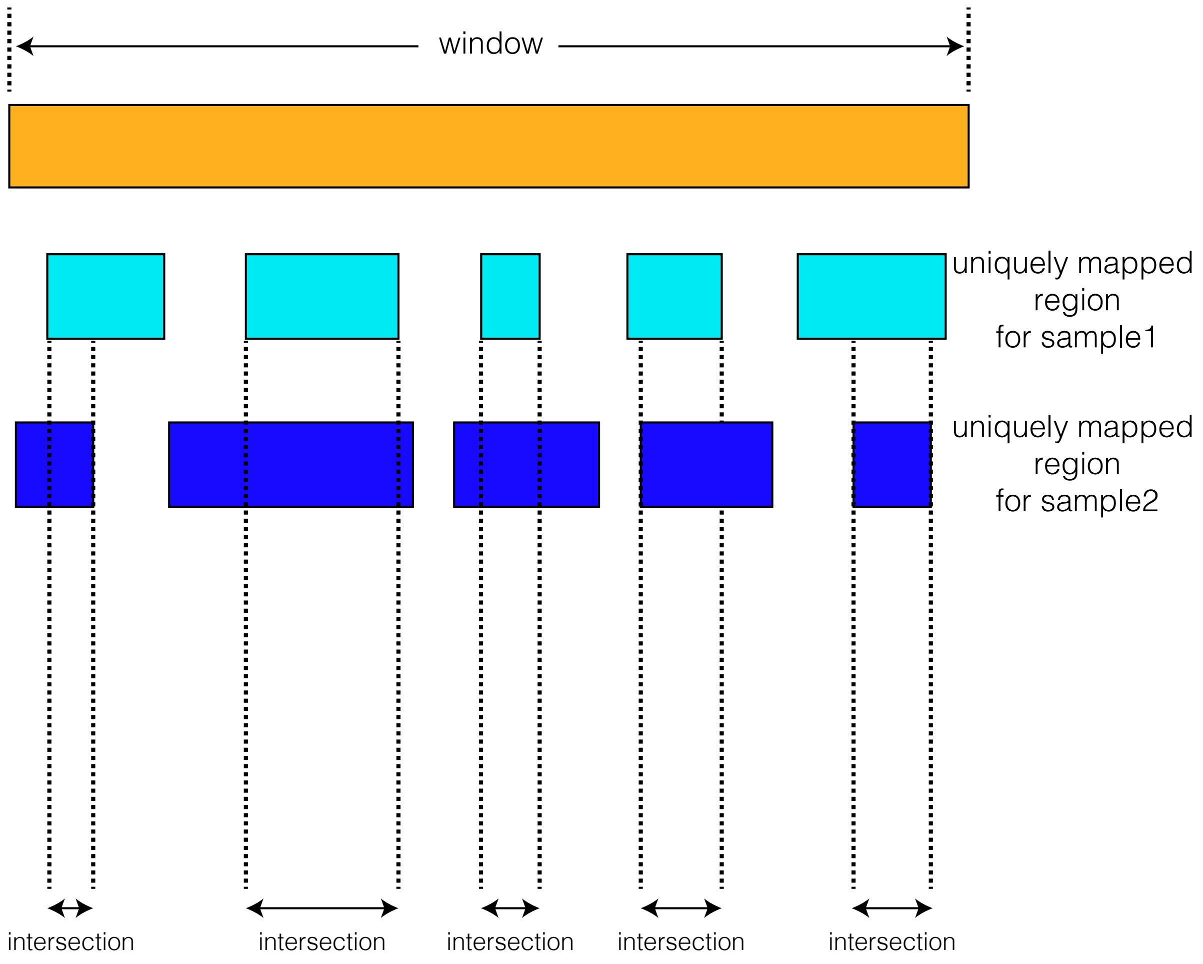

Supplementary Figure 24: Normalization of SNP counts for read depth. To determine the number SNPs between two samples in a given genomic window of size, we first intersect the regions covered by at least two uniquely mapped reads (MAPQ ≥ 20). Then, we calculated the normalized SNP number according to the formula: (raw SNP number / cumulative of the intersected intervals) × window size. The “raw” SNP number was determined with command “sample-diff counts-only” of Plink2.

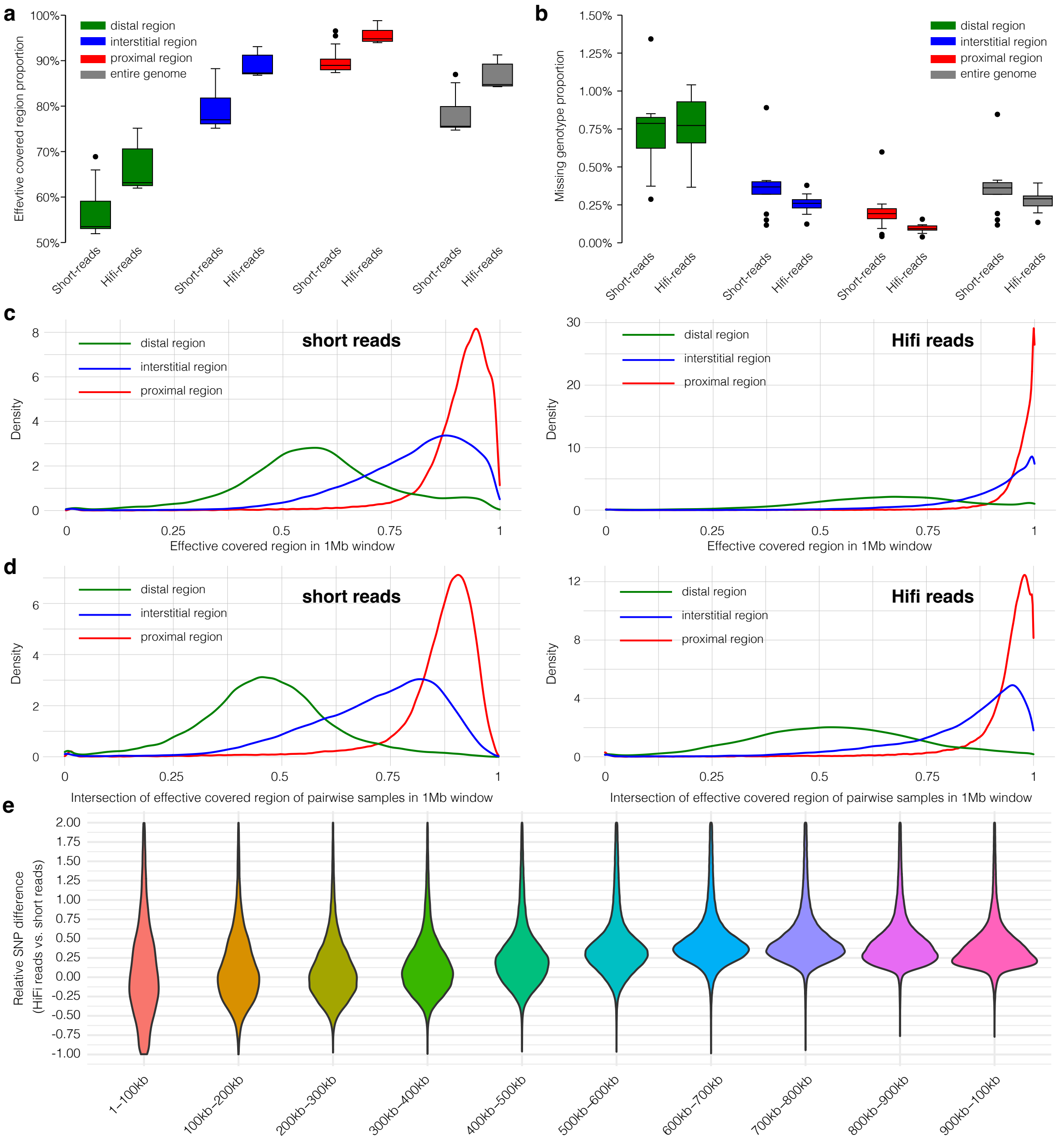

Supplementary Figure 25: Comparison of SNP number estimation using short reads and HiFi reads in three chromosomal regions (distal, interstitial, and proximal) across 13 samples. (a). Comparison of effective covered regions (uniquely mapped regions) between short-read and HiFi-read datasets. **(b)** Comparison of missing genotype rates. For a single sample, missingness is calculated as: number of genotypes filtered to missing / effective covered region. **(c)** Density distribution of the size of effective covered regions. Calculations were performed using 1 Mb windows with a 200 kb step. **(d)** Density distribution of the size of the intersection of effective covered regions between each pair of samples. **(e)** Violin plot of SNP number differences between HiFi reads and short reads in genomic bins grouped by the size of the intersected effective covered region. The bin size is 100 kb. For a given 1 Mb window, the relative SNP difference is defined as: (SNP_number$_{HiFi_reads}$ − SNP_number$_{short_reads}$) / SNP_number$_{short_reads}$. In panels **(a)** and **(b)**, all box plots are based on a sample size n =13. Box plot display the median (center line), the 25th and 75th percentiles

(box bounds), and whiskers that extend to values within 1.5× the interquartile range (IQR); outliers beyond this range are shown as individual points.

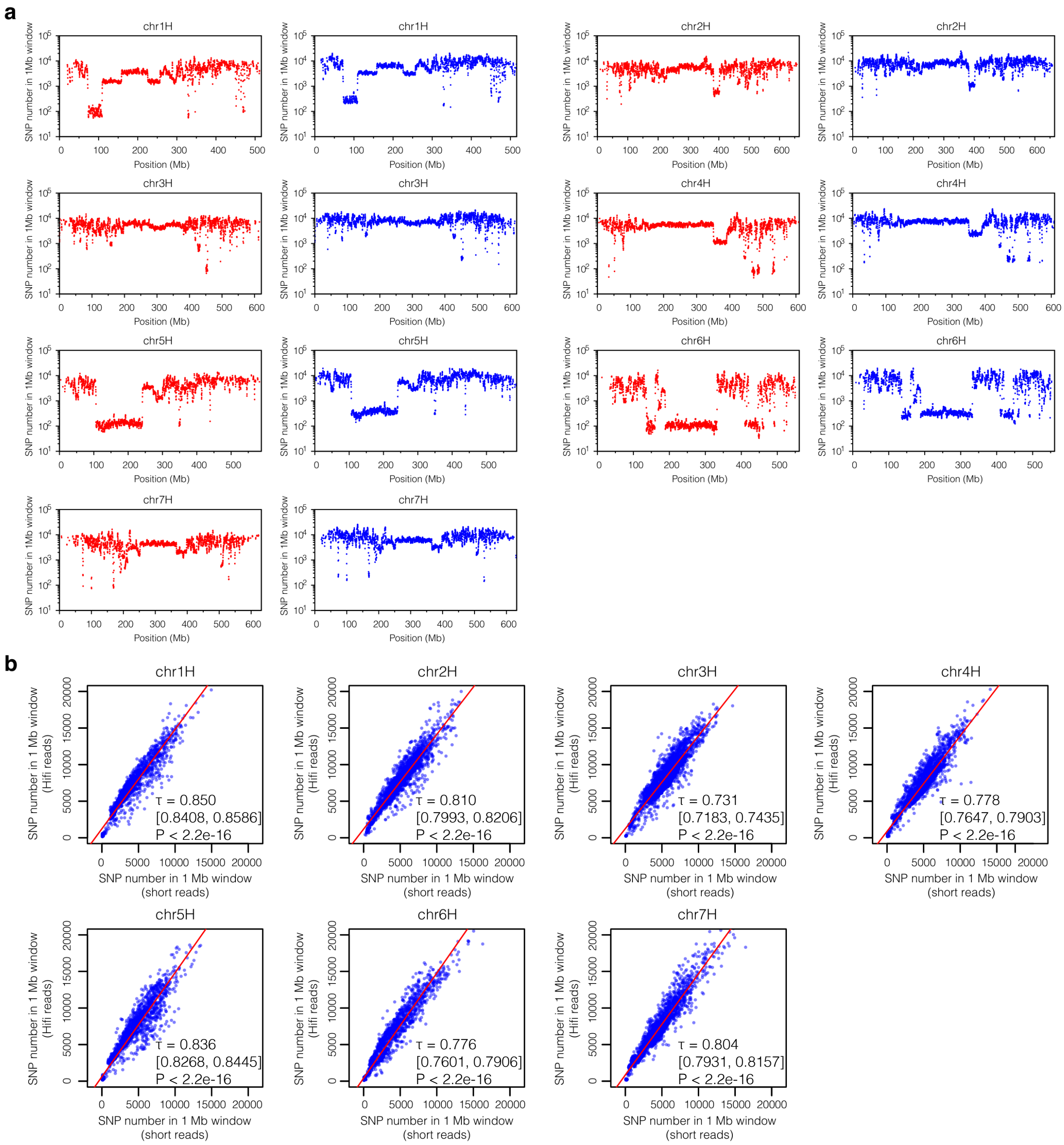

Supplementary Figure 26: Comparison of SNP numbers calculated using short reads and HiFi reads for a sample pair (FT67 vs. WBDC_349) with a 1 Mb sliding window (shift: 200 kb). (a) Distribution of SNP numbers across the seven barley chromosomes. Red dots represent results from short reads, while blue dots represent results from HiFi reads. **(b)** Two-sided Kendall correlation analysis between SNP numbers calculated from short reads and HiFi reads. Since the calculated P-values exceeded the computational lower limit (2.2e-16), we report them as P < 2.2e-16. Values in square brackets denote the 95% confidence interval. For **(a)** and **(b)**, only 1 Mb windows in which the intersection of effective covered regions between the two samples exceeds 0.5 Mb were retained for SNP number calculation.

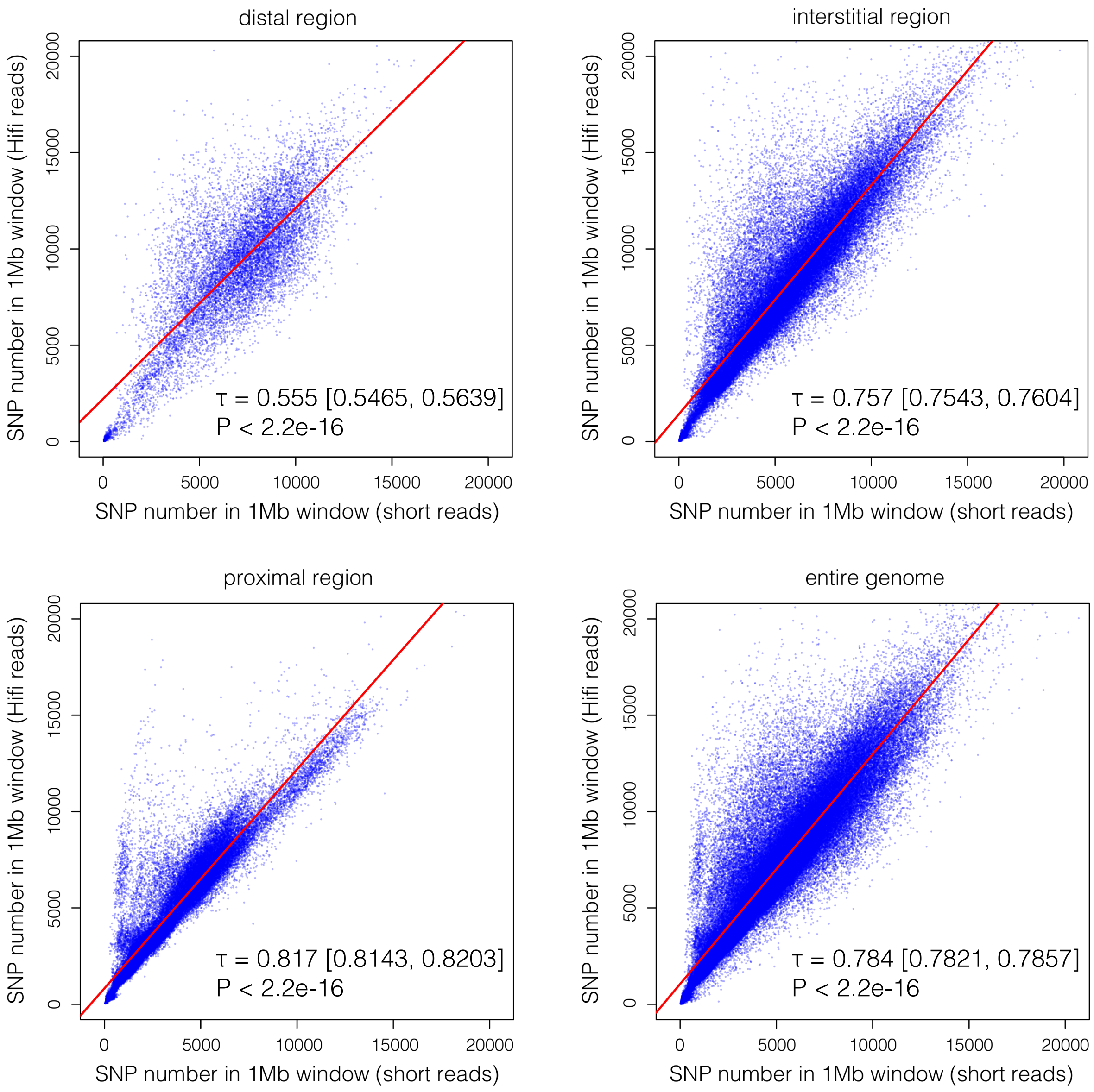

Supplementary Figure 27: Two-sided Kendall correlation of SNP numbers calculated from short reads and HiFi reads across three genomic regions (distal, interstitial, and proximal) for all pairwise comparisons among 13 samples. SNP numbers were computed using a 1 Mb sliding window (shift: 200 kb). Only 1 Mb windows in which the intersection of effective covered regions between the two samples exceeds 0.5 Mb were retained for SNP number calculation. Since the calculated P-values exceeded the computational lower limit (2.2e-16), we report them as P < 2.2e-16. Values in square brackets denote the 95% confidence interval.

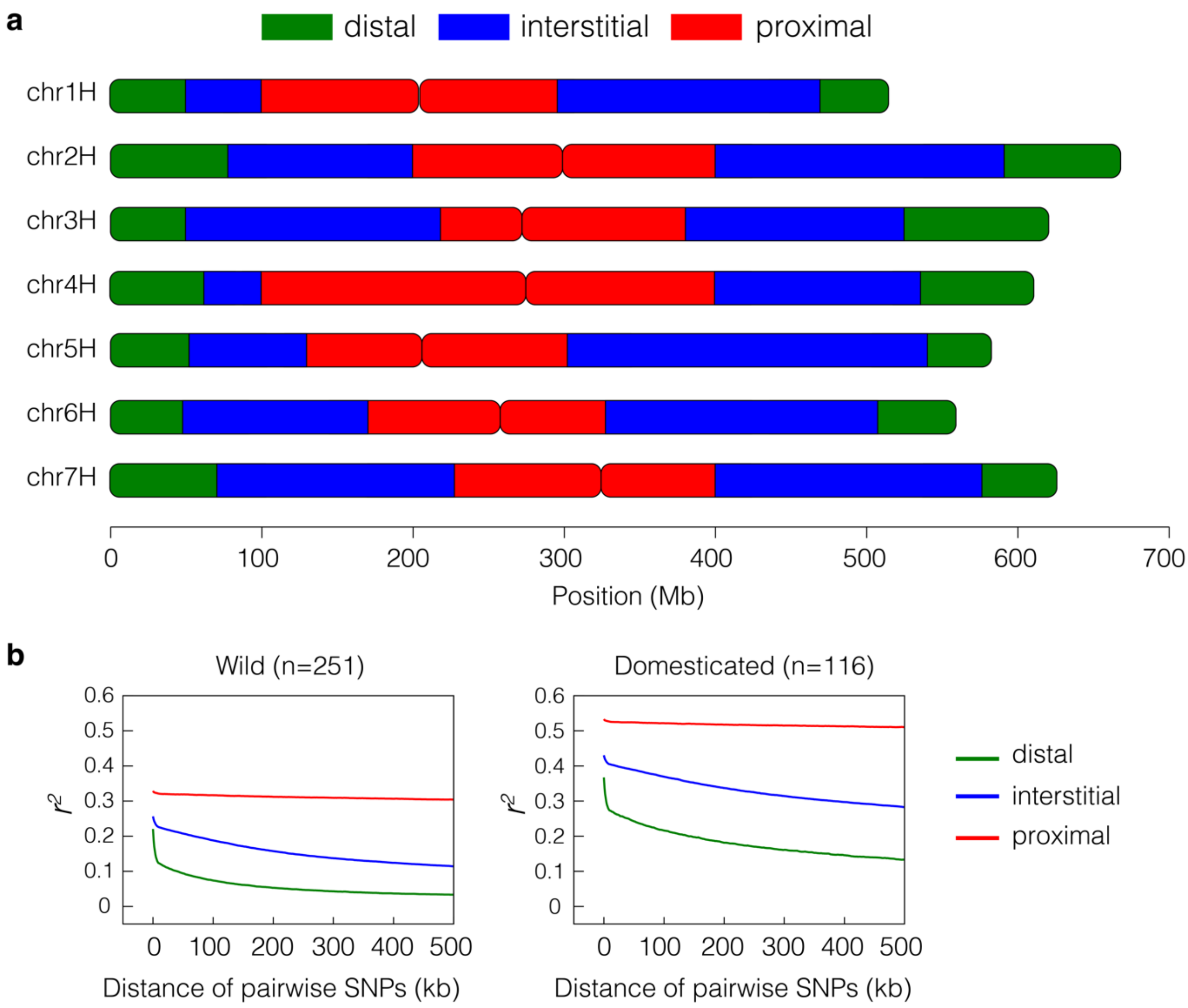

Supplementary Figure 28: Decay of linkage disequilibrium (LD) in three genomic compartments. (a) The distal, interstitial and proximal regions were defined based on differences in recombination rate following Mascher et al. 2017. The precise boundaries of the three compartments of each chromosome are listed in **Supplementary Table 21**. **(b)** LD decay in the three genomic compartments thus defined in wild and domesticated barley.

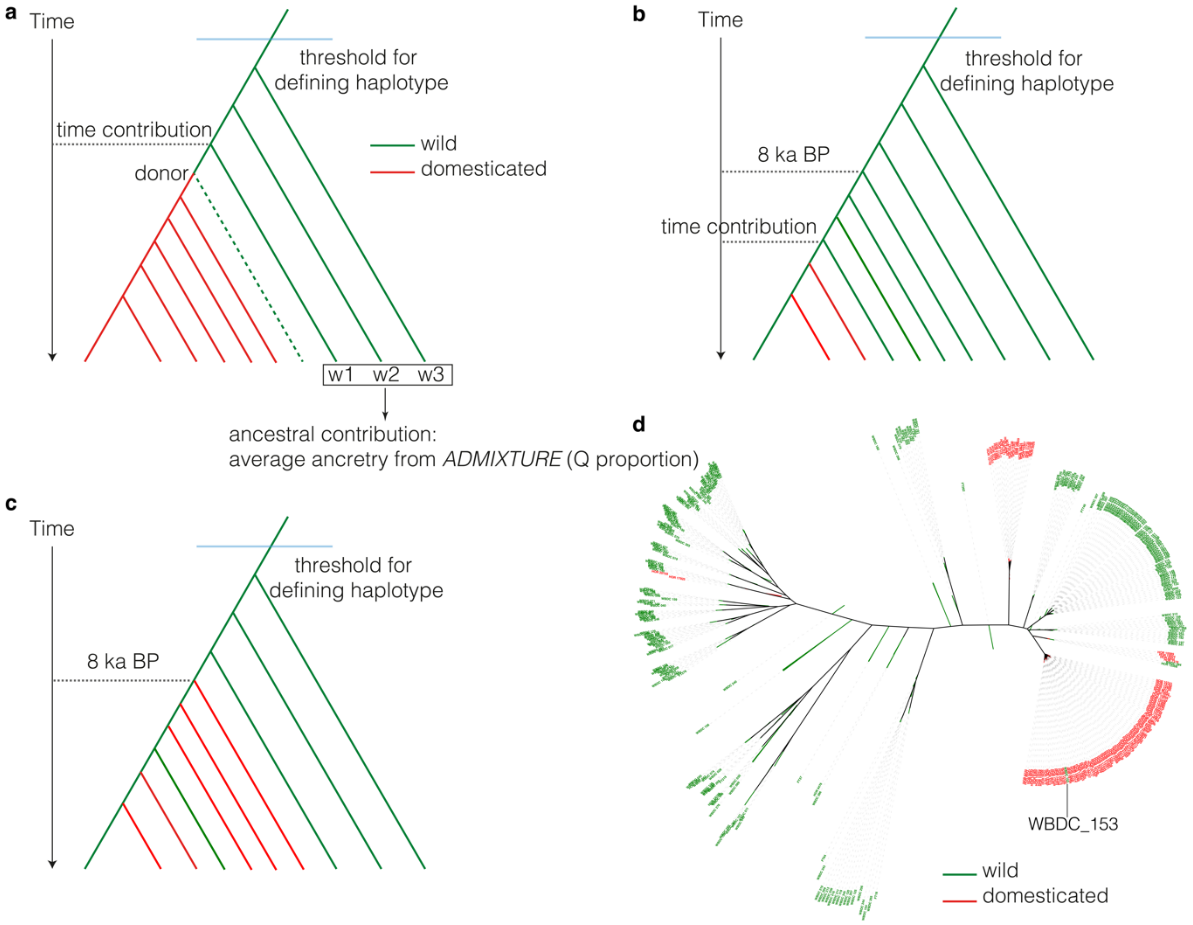

Supplementary Figure 29: Inference of haplotype origins in time and space. Samples in trees have the same haplotype (H) in a genomic region of interest, i.e. their sequence divergence is below a chosen threshold. Red and green color stand for domesticated and wild samples, respectively. **(a)** Dating in the absence of post-domestication introgression. The time of origin of H was set to set time of divergence of the domesticated branch from the closest wild sample (w1). To find the most closely related wild sample, we compared the results of IntroBlocker runs with different thresholds: 400 SNPs (equivalent to an approximate divergence time of 32,000 years ago), 98 SNPs (8,000 years), 73 SNPs (6,000 years), 49 SNPs (4,000 years), and 24 SNPs (2,000 years). To determine the wild source population of population, the ancestry coefficients of all wild barleys (w1, w2, w3) with haplotype H were averaged. In panels **(b)** and **(c)**, the divergence time between domesticated and wild carriers of H is less than 8,000 years, indicating recent gene flow, either in the direction wild > crop **(b)** and crop > wild **(c)**. **(d)**. Neighbor-joining tree constructed from SNPs on chromosome 4H, 250 Mb – 300 Mb. The sample WBDC 153 is the only wild barley that shared a haplotype with domesticated samples. The most likely explanation is geneflow in the direction crop > wild.

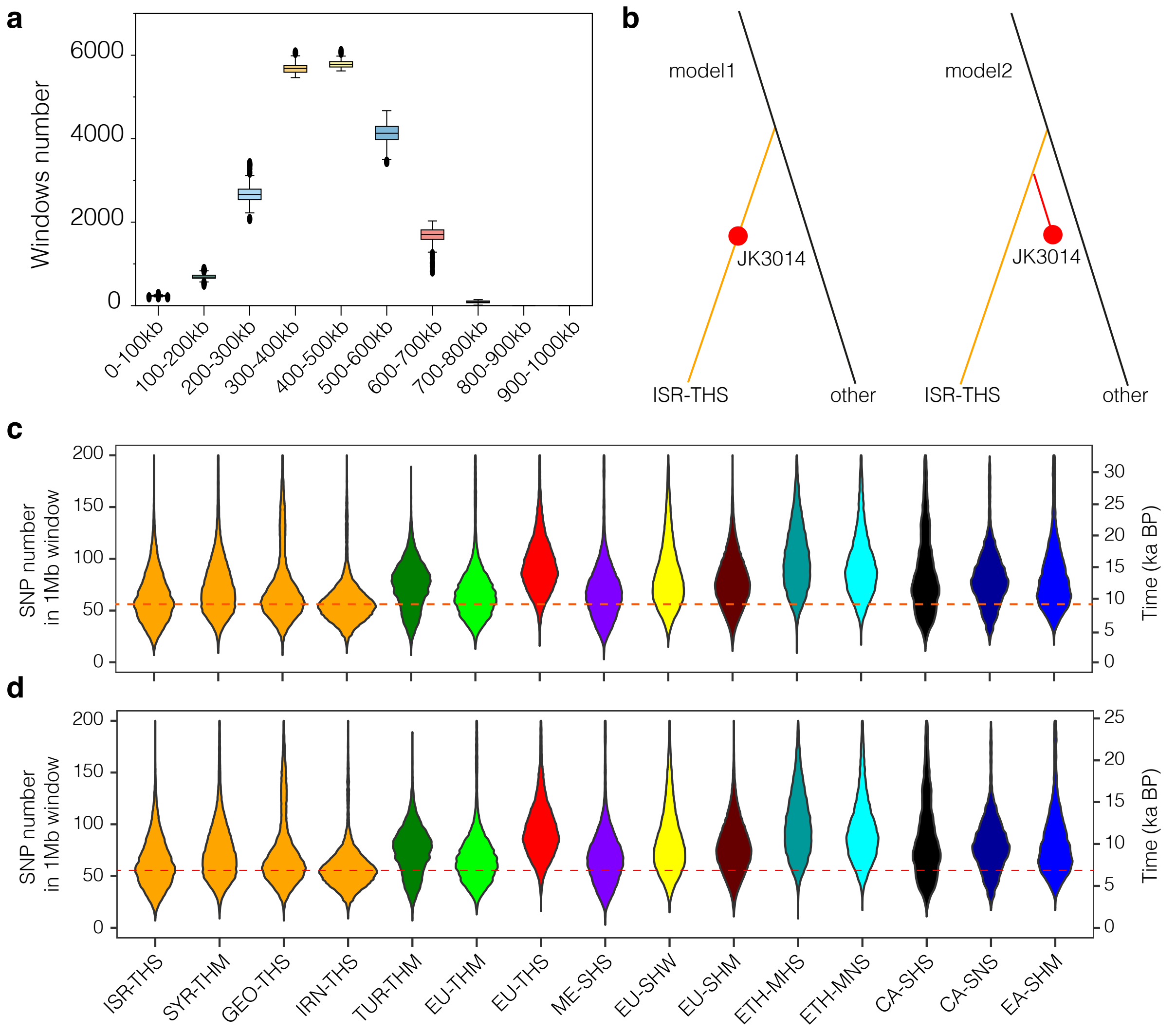

Supplementary Figure 30: Divergence time between the 6,000-year-old two-rowed ancient barley sample JK3014 and modern domesticated barley. SNP number was calculated using 1 Mb sliding windows (shift: 200 kb). **(a)** Window counts of a given effectively covered region intersection bin between JK3014 and each of the 116 modern barley samples. Intersection was binned into 100 kb intervals, and only windows with overlaps greater than 500 kb were retained for downstream analysis. For each box plot, sample pairs having n = 116. Box plots show the median (center line), the 25th and 75th percentiles (box bounds), and whiskers extend to values within 1.5× the interquartile range (IQR); outliers beyond this range are shown as individual points. **(b)** Models describing the divergence between ancient barley (JK3014) and modern Israel two-rowed barley (ISR-THS): model 1 assumes JK3014 is the direct ancestor of ISR-THS; model 2 assumes JK3014 and ISR-THS share a common ancestor, with divergence occurring slightly earlier than 6,000 years ago. **(c)** Violin plot showing the distribution of SNP numbers. The secondary y-axis shows the estimated divergence time under Model 1, calculated as: time = d / μ, which yields an average divergence time of approximately 9,000 years. **(d)** Violin plot showing the distribution of SNP numbers with divergence time estimated under Model 2, using the formula: time = $(d / 1.1) / (1.2 \times \mu)$. This gives an estimated divergence time of around 7,000 years. In **(c)** and **(d)**, the dashed line indicates the SNP number peak, corresponding to the inferred divergence time between ancient and ISR-THS.
