# Supplementary material for: A haplotype-based evolutionary history of barley domestication
Source: Nature. 2025 Sep 24;647(8090):680–8. doi: 10.1038/s41586-025-09533-7 (PMC12629985; doi:10.1038/s41586-025-09533-7)
Supplement: Supplementary file 2 — Reporting Summary [file 41586_2025_9533_MOESM2_ESM.pdf]

nature portfolio

Corresponding author(s): Martin Mascher

Last updated by author(s): Jul 11, 2025

Reporting Summary

Nature Portfolio wishes to improve the reproducibility of the work that we publish. This form provides structure for consistency and transparency in reporting. For further information on Nature Portfolio policies, see our Editorial Policies and the Editorial Policy Checklist.

Statistics

For all statistical analyses, confirm that the following items are present in the figure legend, table legend, main text, or Methods section.

n/a	Confirmed	
☐	☒	The exact sample size (*n*) for each experimental group/condition, given as a discrete number and unit of measurement
☐	☒	A statement on whether measurements were taken from distinct samples or whether the same sample was measured repeatedly
☐	☒	The statistical test(s) used AND whether they are one- or two-sided<br>*Only common tests should be described solely by name; describe more complex techniques in the Methods section.*
☒	☐	A description of all covariates tested
☒	☐	A description of any assumptions or corrections, such as tests of normality and adjustment for multiple comparisons
☐	☒	A full description of the statistical parameters including central tendency (e.g. means) or other basic estimates (e.g. regression coefficient) AND variation (e.g. standard deviation) or associated estimates of uncertainty (e.g. confidence intervals)
☐	☒	For null hypothesis testing, the test statistic (e.g. *F*, *t*, *r*) with confidence intervals, effect sizes, degrees of freedom and *P* value noted<br>*Give P values as exact values whenever suitable.*
☒	☐	For Bayesian analysis, information on the choice of priors and Markov chain Monte Carlo settings
☐	☒	For hierarchical and complex designs, identification of the appropriate level for tests and full reporting of outcomes
☒	☐	Estimates of effect sizes (e.g. Cohen's *d*, Pearson's *r*), indicating how they were calculated

Our web collection on statistics for biologists contains articles on many of the points above.

Software and code

Policy information about availability of computer code

Data collection	Illumina NovaSeq 6000(Illumina Inc., San Diego, CA, USA)
Data analysis	Software used: ADMIXTOOLS (version 3.0), ADMIXTURE (version 1.23), bcftools (version 1.15.1), Beagle (version 5.5), CLUMPP (version 1.1.2), DeepVariant (v1.6.0), Distruct (version 1.1), GEVA (version 1), GLnexus (version 1.3.1), IntroBlocker (version 2), iTOL (version 7), leeHom (version 1.2.17), mapDamage (version 2.0.8), Minimap2 (version 2.24), MUMmer (version 4.0.0), Novosort (version 3.06.05), pbmm2 (v1.10.0), Perl(V5.38.1), PLINK (version 1.9), PLINK2 (version 2.00a3.3LM), PopLDdecay (version 3.42), PSMC (version 0.6.5-r67), R (version 3.5.1), SAMtools (version 1.16.1), smartpca (version 7.2.1), SnpEff (version 4.3t).<br><br>Custom code availability: All custom scripts used in this study are available at https://github.com/guoyu-meng/barley-haplotype-script.

For manuscripts utilizing custom algorithms or software that are central to the research but not yet described in published literature, software must be made available to editors and reviewers. We strongly encourage code deposition in a community repository (e.g. GitHub). See the Nature Portfolio guidelines for submitting code & software for further information.

Data

Policy information about availability of data

All manuscripts must include a data availability statement. This statement should provide the following information, where applicable:

- Accession codes, unique identifiers, or web links for publicly available datasets
- A description of any restrictions on data availability
- For clinical datasets or third party data, please ensure that the statement adheres to our policy

The sequence data collected in this study have been deposited at the European Nucleotide Archive (ENA)79 under BioProjects PRJEB65046, PRJEB56087 and PRJEB53924. The SNP and indel variant matrix are available at the European Variation Archive80 (EVA) under BioProject PRJEB79752. ENA accession codes for individual genotypes are listed in Supplementary Table 1. AHG matrices have been deposited in the Plant Genomics & Phenomics Research Data Repository81 under the DOI: http://doi.org/10.5447/ipk/2025/7.

Research involving human participants, their data, or biological material

Policy information about studies with human participants or human data. See also policy information about sex, gender (identity/presentation), and sexual orientation and race, ethnicity and racism.

Reporting on sex and gender	Not applicable.
Reporting on race, ethnicity, or other socially relevant groupings	Not applicable.
Population characteristics	Not applicable.
Recruitment	Not applicable.
Ethics oversight	Not applicable.

Note that full information on the approval of the study protocol must also be provided in the manuscript.

Field-specific reporting

Please select the one below that is the best fit for your research. If you are not sure, read the appropriate sections before making your selection.

☐ Life sciences ☐ Behavioural & social sciences ☒ Ecological, evolutionary & environmental sciences

For a reference copy of the document with all sections, see nature.com/documents/nr-reporting-summary-flat.pdf

Ecological, evolutionary & environmental sciences study design

All studies must disclose on these points even when the disclosure is negative.

Study description	The evolutionary history of barley domestication.
Research sample	380 wild barley and 302 domesticated barley accessions.
Sampling strategy	Selection based on preliminary population structure
Data collection	Genebank accessions, DNA sequencing
Timing and spatial scale	Not applicable
Data exclusions	Admixed samples were excluded from downstream analysis as described in the manuscript.
Reproducibility	WGS data were compared with genebank genomics GBS data to confirm sample identities.
Randomization	Not applicable.
Blinding	Not applicable.

Did the study involve field work? ☐ Yes ☒ No

Reporting for specific materials, systems and methods

We require information from authors about some types of materials, experimental systems and methods used in many studies. Here, indicate whether each material, system or method listed is relevant to your study. If you are not sure if a list item applies to your research, read the appropriate section before selecting a response.

Materials & experimental systems

n/a	Involved in the study
☒	☐ Antibodies
☒	☐ Eukaryotic cell lines
☐	☒ Palaeontology and archaeology
☒	☐ Animals and other organisms
☒	☐ Clinical data
☒	☐ Dual use research of concern
☐	☒ Plants

Methods

n/a	Involved in the study
☒	☐ ChIP-seq
☒	☐ Flow cytometry
☒	☐ MRI-based neuroimaging

Palaeontology and Archaeology

Specimen provenance	We analyzed ancient DNA sequences of 23 barley grains excavated at three archaeological sites in Israel (Supplementary Table 11). This number included published data of 5 barley grains from Yoram Cave. Archaeobotanical procedures were performed as described by LevMarom et al. Yoram Cave and Timna Valley Site have been described by Mascher et al. and Lev-Marom et al. Abi'or Cave is a medium-sized cave located on the eastern slopes of the Judean Desert, above Jericho, approximately 50 meters below sea level, across from the Karantal Monastery. The excavaEons at the cave were directed by the late H. Eshel in 1986. It is situated above a larger cave known as "The Spies Cave". The cave contains a main long tunnel, approximately 50 meters long, and has revealed archaeological material daEng from the Chalcolithic period to the Eme of the Bar Kokhba Revolt (2nd century CE). The cave was found to be heavily disturbed by animals, anEquiEes robbers, and monks who lived in it during the Islamic and more recent periods.
Specimen deposition	Yoram Cave, Timna Valley Site 34 and Abi'or Cave.
Dating methods	Radiocarbon dating of single seeds

☒ Tick this box to confirm that the raw and calibrated dates are available in the paper or in Supplementary Information.

Ethics oversight	Not applicable

Note that full information on the approval of the study protocol must also be provided in the manuscript.

Plants

Seed stocks	Leibniz Institute of Plant Genetics and Crop Plant Research (IPK)
Novel plant genotypes	Not applicable
Authentication	WGS data were compared with genebank genomics GBS data to confirm sample identities.
